# The Classification for Adverse Events in GastRointEstinal Endoscopy (AGREE): Three-Year Evaluation and Additional Recommendations

**DOI:** 10.1055/a-2965-6729

**Published:** 2026-10-13

**Authors:** Querijn N.E. van Bokhorst, Hessel Bierma, Karlijn J. Nass, Evelien Dekker, Manon van der Vlugt, Paul Fockens

**Affiliations:** 1 Department of Gastroenterology and Hepatology 1209 Amsterdam UMCAmsterdamthe Netherlands; 2 571165 Amsterdam Gastroenterology Endocrinology MetabolismAmsterdamthe Netherlands; 3 Cancer Center AmsterdamAmsterdamthe Netherlands; 4 Department of Gastroenterology and Hepatology 1322 Rijnstate HospitalArnhemthe Netherlands; 5 Department of Gastroenterology and Hepatology 1209 Waikato HospitalHamiltonNew Zealand

**Keywords:** quality and logistical aspects, performance and complications, quality management

## Abstract

**Background** In 2021, a novel classification for gastrointestinal endoscopy-related adverse events (AEs) was introduced: the AGREE classification. This classification was developed to minimize subjectivity in AE assessment and facilitate more robust comparisons of endoscopic and surgical procedures. We conducted a comprehensive review of the classification three years after its introduction.

**Methods** A citation analysis of the original AGREE publication was performed through March, 2025. Identified articles were reviewed to evaluate the classification’s adoption, application, use for intra- and interdisciplinary comparisons, and comparability with other classifications. Accuracy of AE classifications was assessed based on review of unique scenarios reported across identified articles. Variability in the definition and reporting of AEs across studies was evaluated.

**Results** In total, 167 unique articles, involving 33 countries across 5 continents, were identified. A notable year-over-year increase was observed. Available studies illustrated feasibility of the AGREE to facilitate both intra- and interdisciplinary comparisons, alongside significant correlations between AGREE-based AE severity assessments and assessments using other classifications. Accuracy of gradings was estimated at 85%. Misclassifications mostly involved overclassification of events without sequelae for the patient. Inconsistencies in grading, definition, and reporting of AEs were identified. Findings were used to establish recommendations for grading specific (challenging) AE scenarios and develop a checklist to support more standardized AE reporting.

**Conclusions** A gradual increase in the annual citations of the AGREE classification was observed, suggesting growing awareness and academic interest. The recommendations provided could support more consistent grading, definition, and reporting of AEs, thereby improving opportunities for endoscopy quality evaluation.

## Introduction

Gastrointestinal (GI) endoscopy is the primary method for diagnosing and treating diseases of the GI tract. Despite its clinical value, endoscopy carries a risk of adverse events (AEs), particularly when performing therapeutic (interventional) procedures. Standardized reporting and evaluation of endoscopy-related AEs and their sequelae is crucial to monitor the safety of endoscopic procedures and allow for robust comparisons of clinical outcomes across various (endoscopic) techniques, patient groups, endoscopists, and institutions. 1 2 3 4

In December 2021, we proposed the AGREE classification as a novel classification for grading AEs related to GI endoscopy. 5 This classification was developed to minimize subjectivity in AE assessment by addressing the two major limitations of the widely used American Society for Gastrointestinal Endoscopy (ASGE) classification. 6 Primarily, the AGREE classification eliminated the use of subjective descriptors such as “mild,” “moderate,” and “severe” when reporting the severity of AEs. Besides, the AGREE classification explicitly focuses on clinical consequences of AEs, rather than on factors that could also be affected by social or logistic issues (e.g., hospital stay duration). Moreover, as the AGREE classification is adapted from the surgical Clavien–Dindo classification, 7 8 it was designed to allow for easier and more robust comparisons of clinical outcomes between endoscopic and surgical interventions.

We conducted a comprehensive review of the AGREE classification three years after its introduction. This review aimed to the evaluate the adoption and application of the classification, as well as its comparability to other classifications. Moreover, we aimed to evaluate the accuracy of AE grading using the AGREE classification based on (potential) AE scenarios reported across studies. Lastly, we sought to identify inconsistencies in grading, definition, and reporting of AEs. Findings were used to develop additional recommendations for the grading and reporting of AEs.

## Methods

### Methodological Framework

A citation analysis of the original article introducing the AGREE classification was performed. 5 9 The adoption and application of the classification were evaluated based on trends in annual citations, global spread, fields of application, and its use for intra- and interdisciplinary AE comparisons. Moreover, we aimed to obtain insights into the comparability of the AGREE classification with other classifications by summarizing evidence from studies evaluating the correlation between AGREE-based AE severity assessments and assessments using other (longer-established) classifications. Furthermore, unique AE scenarios were extracted from identified articles with the aim to evaluate the accuracy of AE grading using the AGREE classification, as well as to identify AE scenarios for which severity grading could be considered challenging. Challenging scenarios were discussed among the authors of the original AGREE publication, and expert opinion-based recommendations for classification of these scenarios were established. Finally, the way in which AEs were defined and reported across identified articles was reviewed. Findings were used to develop a checklist to support more standardized AE reporting.

Given the bibliometric (citation-based) nature of this review, it was not prospectively registered in a public systematic review database.

### Eligibility Criteria and Search Strategy

All articles written in English and referencing the original article introducing the AGREE classification 5 were considered eligible for inclusion. We identified articles referencing this article by using the “cited by” functionality of the MEDLINE (PubMed), Web of Science, SCOPUS, and Google Scholar databases. Databases were searched for articles published between December 8, 2021 (publication of the original article) and March 1, 2025. Reference screening was performed to identify additional relevant articles. The database search and reference list screening were performed by two authors independently (Q.B. and H.B.).

### Data Extraction and Evaluation

Identified articles were divided into “original research” articles, defined as articles presenting new data, findings, or insights generated by the authors through first-hand (clinical) investigation or experimentation, and “other” articles (e.g., review articles). All articles were screened by two authors (Q.B., H.B.), and relevant data was independently extracted using structured tables ( **Tables S1–S3** , available in the online version). In case of any discrepancies in extracted data, these were resolved through discussion.

Trends in annual citations, the application of the AGREE classification for intra- and interdisciplinary comparisons, and the correlation between AE severity gradings based on both the AGREE and other classifications were evaluated based on data derived from both *original research* and *other* articles. The global spread (i.e., countries involved in the identified papers) and fields of application (i.e., type of endoscopic procedures) were evaluated based on data derived from *original research* articles only. Similarly, the evaluation of AE scenarios and AE reporting practices was performed including *original research* articles only. However, studies that cited the original AGREE publication 5 without actually applying the AGREE classification for AE grading were excluded from these analyses.

To evaluate the accuracy of AE gradings using the AGREE classification, all potential AE scenarios reported across identified *original research* articles for which the type of event, consequences for the patient, and a specific AGREE classification grade were available, were independently extracted and reviewed for correct grading by two authors (Q.B., H.B.). Any scenario for which severity grading was considered challenging by at least one of reviewers, or for which the assigned grade was considered potentially incorrect by at least one of the reviewers, was labeled a “challenging scenario.” All *challenging scenarios* were discussed in a consensus meeting involving the two independent reviewers and three of the lead authors (K.N., M.V., P.F.) of the original AGREE article aimed at establishing consensus on the correct classification of these scenarios. 5 Any disagreements in the interpretation of the scenarios were resolved through discussion until agreement was reached among all participants. Eight of the discussed scenarios, along with recommendations on how to interpret and grade the corresponding (adverse) events, were selected to draft illustrative scenarios for discussion in this study. A single fictional scenario was added based on the authors’ discretion.

Variability in the definition and reporting of AEs was evaluated based on the following criteria: (1) reporting of the AE definition(s) used; (2) reporting of the classification(s) used; (3) reporting of the timeframe during which AEs were monitored; (4) the extent to which grade III and IV AEs were adequately subdivided using suffixes (e.g., IIIa vs. IIIb); and (5) the use of subjective terminology related to both the severity and timing of (the of onset) AEs. A checklist to support more standardized AE reporting was developed by adapting existing AE reporting checklists and recommendations 10 11 12 13 and integrating the specific findings of this study.

### Statistical Considerations

The accuracy of AE grading across studies was defined as the percentage of AEs for which the assigned AGREE severity grade was deemed incorrect after review, with the grading determined in the consensus meeting serving as the reference standard. Cases where only a suffix was missing (e.g., “III” instead of “IIIa”), or a conclusion regarding accuracy of the classification could not be drawn due to insufficient information, were not considered misclassifications. If multiple events within one article yielded similar characteristics (in terms of the type of event, consequences for the patient, and AGREE grade), these were considered a single AE scenario for the analyses performed.

## Results

### Trends in Annual Citations, Global Spread, and Fields of Application

A total of 167 unique articles were identified through the citation analysis. Reference screening did not result in identification of additional articles ( Fig. 1 ). In terms of the number of annual citations, a notable year-over-year increase was observed ( Fig. 2 ). Of the 167 identified records, 113 involved *original research* articles. These were conducted across 33 countries spanning 5 continents ( Fig. 3 ). The majority of identified *original research* articles (100/113; 88%) focused on interventional endoscopy, mostly involving the hepato-pancreatico-biliary (49/113; 43%) or upper GI (42/113; 37%) tract. More detailed insights into the study design, involved countries, and areas of application for identified articles are reported in **Table S1** and **Fig. S1** (available in the online version).

**Fig. 1:**
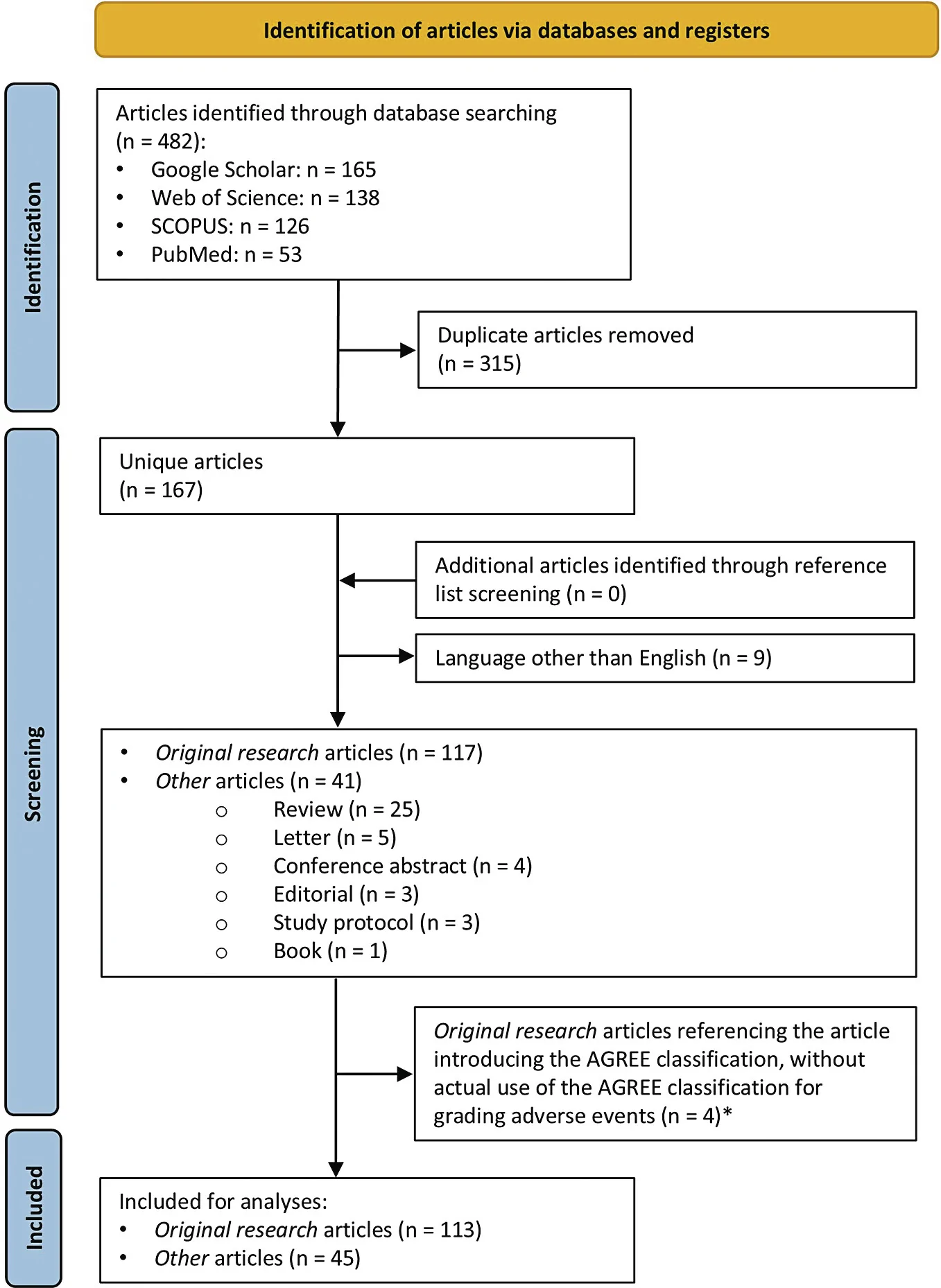
Study flowchart. *Original research articles referencing the original article introducing the AGREE classification, without the actual use of the AGREE classification to grade severity of adverse events (n = 4), were allocated to the “other” articles group.

**Fig. 2:**
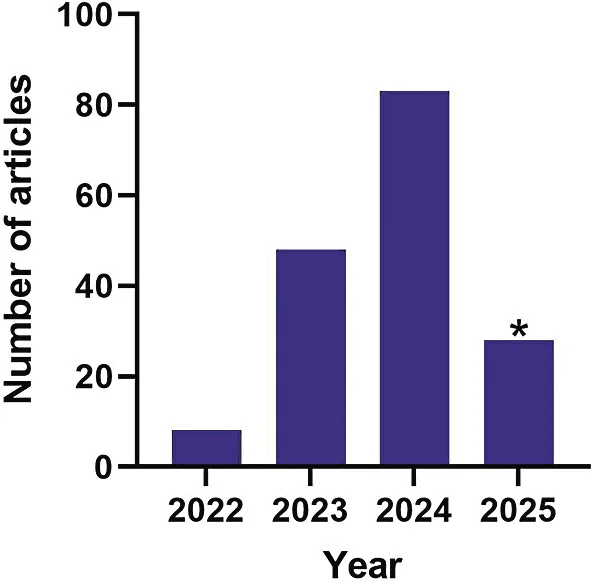
Annual number of articles citing the AGREE classification. *Data for 2025 only include the months January and February.

**Fig. 3:**
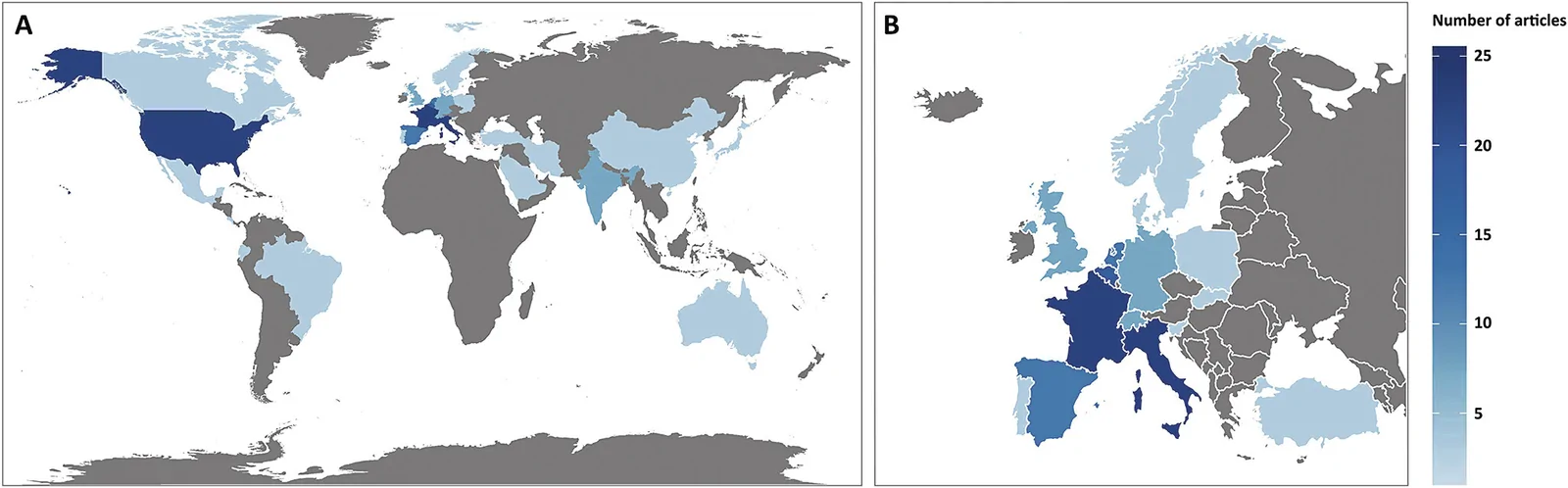
Geographic distribution of original research articles using the AGREE classification to grade adverse events related to gastrointestinal endoscopy: ( **A** ) global, ( **B** ) Europe.

### Use for Intra- and Interdisciplinary Comparisons of Adverse Events

In terms of intradisciplinary comparisons, 22 of the identified *original research* articles specifically used the AGREE classification to compare the severity of AEs across different endoscopic treatment modalities within similar patient subgroups. 14 15 16 17 18 19 20 21 22 23 24 25 26 27 28 29 30 31 32 33 34 35 Another 11 articles applied the AGREE classification to compare the severity of AEs for similar procedures across varying patient populations, or among endoscopists with different levels of expertise or professional backgrounds. 35 36 37 38 39 40 41 42 43 44 45 One study used the classification to compare AEs between institutions. 46 Additionally, we identified two articles that used the AGREE classification to aggregate and compare severities of AEs across endoscopic procedures. 47 48

In terms of interdisciplinary comparisons, three studies evaluated endoscopic and surgical treatment approaches for specific GI disorders, utilizing both the AGREE and Clavien–Dindo classifications to grade endoscopy- and surgery-related AEs, respectively. 49 50 51 Another study used the AGREE classification to facilitate the comparison of (severity of) AEs associated with both endoscopic and radiology-guided interventions. 52 Although outside the primary scope of this study, we also identified several study protocols (NCT06038201, NCT05745012, NCT04330846) with the documented intention to use both the AGREE and Clavien–Dindo classifications to compare (outcomes of) endoscopic and surgical interventions.

### Correlation with Other Classifications

Similar to the original AGREE publication, 5 two studies evaluated the correlation between the severity of AEs as assessed using both the AGREE and ASGE classifications. A study by Katrevula et al, 53 involving 220 AEs, found a significant positive correlation between AE severities as assessed using both classifications (Pearson correlation coefficient = 0.79; *P* < 0.001). Similarly, a study by Crispino et al, 54 involving 226 AEs, found a moderate positive correlation (ρ = 0.61) alongside a moderately significant association (Cramer’s *V* = 0.7; *p* < 0.01). The aforementioned study also assessed inter-observer agreement for AE grading, demonstrating higher concordance among clinicians using the AGREE classification compared to the ASGE classification (Cohen’s κ: 0.80 vs. 0.60). A study by Yvamoto et al. compared AE severities according to both the AGREE and Clavien–Dindo classifications, reporting perfect agreement between both classifications in the identification of “serious” AEs (grade > III AEs according to both classifications). 55

### Accuracy of Adverse Event Classification and Challenging Scenarios

A total of 179 unique AE scenarios were extracted from *original research* articles ( **Table S3** , available in the online version). Of these, 26 scenarios (15%) were considered misclassifications after review. The majority of these misclassifications (20 out of 26, 77%) were attributable to “overclassification”: events graded as AEs despite the absence of sequelae for the patient, thus not meeting the AGREE criteria for an AE ( **Table S4** , available in the online version).

A summary of the *challenging scenarios* selected for discussion in this study, along with the expert opinion-based recommendations regarding their grading, is available in Table 1 . A more detailed description and appraisal of these scenarios is available in **Appendix S1** (available in the online version). To address the issue of overclassification, Table 1 includes specific recommendations to document (unforeseen) nonconsequential events as “incidents” when their documentation is considered relevant despite the absence of significant patient sequelae. This may involve documentation for purposes such as the evaluation of novel or emerging endoscopic techniques, assessing endoscopists’ proficiency, or determining the eligibility of specific patient groups to undergo certain procedures.

**Table 1:** Scenarios describing (potential) adverse events along with expert opinion-based recommendations on how to grade these events using the AGREE classification.

Scenario	Severity	Summary of adverse event scenario	Evaluation and consensus-based recommendation
** 1. Intraprocedural event not requiring intervention, without sequelae **	No AE	Superficial jejunal mucosal lacerations observed during motorized spiral enteroscopy. No intervention required, no deviation from the standard postprocedural course.	As there were no sequelae for the patient or deviations from the standard postprocedural course, this event does not meet the AGREE classification criteria for an AE *.
** 2. Intraprocedural AE requiring (additional) intervention, without sequelae **	No AE	Inadvertent LAMS deployment into the peritoneal cavity during EUS-guided choledochoduodenostomy. Immediate retrieval and successful placement of a second LAMS during the same session.	Although an additional intervention was required, there were no sequelae for the patient or deviations from the standard postprocedural course. Therefore, this event does not meet the AGREE classification criteria for an AE *.
** 3. Intraprocedural event requiring (additional) intervention, with sequelae **	Grade I	Sigmoid intussusception during withdrawal of a motorized spiral enteroscope, resolved through parallel insertion of a pediatric colonoscope during the same session. Patient was admitted for observation (<24 h).	As additional observation (<24 h) was required, this event qualifies as a grade I AE . This event does not specifically qualify as a grade IIIa AE as no additional endoscopic procedure ^†^ was required.
**4. Event occurring during a follow-up procedure (within 30 days following the initial procedure)**	Grade IIIb	EUS-guided gastro-gastrostomy to facilitate ERCP in a patient with a history of a gastric bypass. During the ERCP, performed eight days following the gastrostomy, LAMS dislocation led to a gastric perforation requiring surgical intervention.	Although the event was contingent on the prior gastro-gastrostomy, the initial procedure was uneventful. As surgery was required, this event qualifies as a grade IIIb AE associated with the ERCP.
**5. Event related to dysfunction of endoscopically inserted materials**	Grade IIIa	EUS-guided gallbladder drainage for acute cholecystitis using a LAMS. Recurrent cholecystitis due to food-related LAMS obstruction 11 days following the procedure. Repeat endoscopy with balloon sweeping and double-pigtail stent placement to resolve obstruction.	Although (food-related) stent occlusion may be considered unavoidable, early occlusion can reflect suboptimal stent placement or positioning. Therefore, all material-related events with an onset within 30 days should be considered an AE if associated with sequelae for the patient. As repeat endoscopy was required, this event qualifies as a grade IIIa AE ^‡^ .
**6. Intraprocedural event resulting in unscheduled intervention crossover**	Grade II	EUS-guided drainage of walled-off necrosis, intended to be performed using a plastic stent (for study purposes). Significant intraprocedural bleeding, resulting in decision to crossover to drainage using a metal stent. Intraprocedural management of the bleeding. Requirement of postprocedural blood transfusion.	Alterations to the standard postprocedural course were induced by the intraprocedural bleeding. As blood transfusion was required, this event qualifies as a grade II AE . While the bleeding also resulted in unscheduled intervention crossover, it was not the primary cause of the sequelae for the patient ^§^ .
**7. Detection of abnormalities during a (routine) follow-up procedure**	No AE	Endoscopic drainage of a pancreatic fluid collection using a LAMS. Migration of LAMS observed during a routine (scheduled) follow-up endoscopy (<30 days). The patient was asymptomatic. Removal of the LAMS and uneventful deployment of a new LAMS.	As the stent migration was detected during a routine (scheduled) follow-up endoscopy and there were no sequelae for the patient, this event does not meet the criteria for an AE according to the AGREE classification*.
**8. Event with an onset within 30 days and evolving clinical consequences beyond 30 days**	Grade V	Esophageal perforation due to balloon dilatation. Repeat endoscopy for placement of a fully covered stent several hours after the initial procedure. Development of an esophageal-respiratory fistula and heart failure (becoming apparent >30 days after the procedure), which caused death 53 days following the dilatation.	While the fistula became apparent more than 30 days after the procedure, it was directly related to an event (perforation) with an onset within 30 days. When multiple AEs occur after the same endoscopy and are clearly related to each other (e.g., perforation and fistula), the most severe event should be considered. Accordingly, as this event resulted in death of the patient, it qualifies as a grade V AE.
**9. Event potentially related to a scheduled endoscopic procedure** ^¶^	Grade V	Patient scheduled for a routine surveillance colonoscopy. Occurrence of a cardiac arrest during in-hospital preparation for colonoscopy, resulting in death of the patient.	According to the AGREE recommendations, AEs should be recorded in an intention-to-scope manner. Thus, AEs occurring during procedure preparation should also be reported. As this event occurred during colonoscopy preparation and resulted in death of the patient, this event qualifies as a grade V AE.

Abbreviations: AE, adverse event; EUS, endoscopic ultrasonography; LAMS, lumen-apposing metal stent; ERCP, endoscopic retrograde cholangiopancreatography.

*Although such events do not meet criteria for an AE according to the AGREE classification, documentation of unforeseen, nonconsequential events may still be essential when assessing the safety profile of novel or emerging endoscopic techniques. Moreover, documenting such events may prove useful in evaluation of endoscopists’ proficiency in specific procedures and techniques, or eligibility of selected patient groups to undergo certain procedures. Accordingly, in line with the ASGE recommendations, 6 we recommend to document relevant events not qualifying as AEs as “incidents.”

^†^ This refers to endoscopic procedures that had to be additionally scheduled (at another timeslot or day), and does specifically not refer to additional endoscopic interventions that are performed during the same session.

^‡^ In case the timeframe during which AEs were monitored exceeds 30 days, we recommend to document any (relevant) material-related events occurring after 30 days as “incidents” (see * for rationale). This recommendation is based on the decreasing likelihood that AEs occurring after 30 days are directly attributable to the (quality of the) endoscopic procedure initiated for material insertion. Instead, such events are more likely to reflect factors related to material performance, such as patency, and thus primarily offer insight into the long-term durability of the treatment or device, rather than the risk associated with the endoscopic procedure.

^§^ Decision for intervention crossover was driven by the intraprocedural bleeding. While the intervention crossover did not cause any sequelae for the patient or deviations from the standard post-procedural course, we recommend to document such events as “incidents” (see * for rationale).

^¶^ This event scenario was not based on an AE scenario identified within the literature but involves a fictional case.

### Variability in the Definition and Reporting of Adverse Events

Considerable inconsistencies in the definition and reporting of AEs were observed across studies. Primarily, several articles applied the AGREE classification to grade AE severity, while adhering to an AE definition other than the definition proposed with the AGREE classification ( **Tables S2** and **S5** , available in the online version). 19 24 26 56 57 58 59 60 61 62 63 64 65 66 67 68 Moreover, only 52 out of 113 (46%) *original research* articles reported the timeframe during which AEs were monitored. Among these, several employed a follow-up timeframe other than the 30-days postprocedural period recommended with the AGREE classification ( **Table S2** , available in the online version). 5 In most cases, no rationale for adoption of a divergent follow-up duration was provided.

We also identified various factors that could hinder adequate interpretation or comparison of the severity of reported AEs. In some cases, this pertained to a lack of, or inconsistency in, the specification of AE severity using AGREE-specific grades (e.g., “grade I”, “grade II”, etc.). 33 69 70 71 72 73 74 75 76 77 Moreover, in 15 out of 57 (26%) articles reporting grade III and/or IV AEs, grade IIIa and IIIb and/or grade IVa and IVb AEs were not distinguishable due to the lack of suffixes ( **Table S2** , available in the online version). Furthermore, various articles used subjective terminology in relation to both the severity (47 out of 133 articles; 35%) and timing (32 out of 133 articles; 24%) of AEs ( **Table S2** , available in the online version). The used terminology in relation to severity and timing of observed AEs was provided within the methods section in 17 out of 47 (36%) and 20 out of 32 (63%) articles, respectively.

We also identified considerable heterogeneity in the definition of used terminology. For example, grade II AEs were referred to as “minor,” “mild,” “mild-to-moderate,” “clinically relevant,” “non-severe,” “severe,” “serious,” and “significant” across articles ( Fig. 4A, B ; **Table S2** [available in the online version]). In terms of terminology related to timing of (the onset of) AEs, an example relates to the definition of “early AEs”: these were defined as AEs occurring either within 24 h, 19 30 36 78 79 48 h, 42 64 80 81 one week, 24 82 two weeks, 83 or four weeks 66 following the procedure. Additionally, one article defined “early AEs” as AEs occurring during hospitalization, 84 while another article considered specific subtypes of events (e.g., pancreatitis, bleeding) “early AEs” 85 ( **Table S2** , available in the online version).

**Fig. 4:**
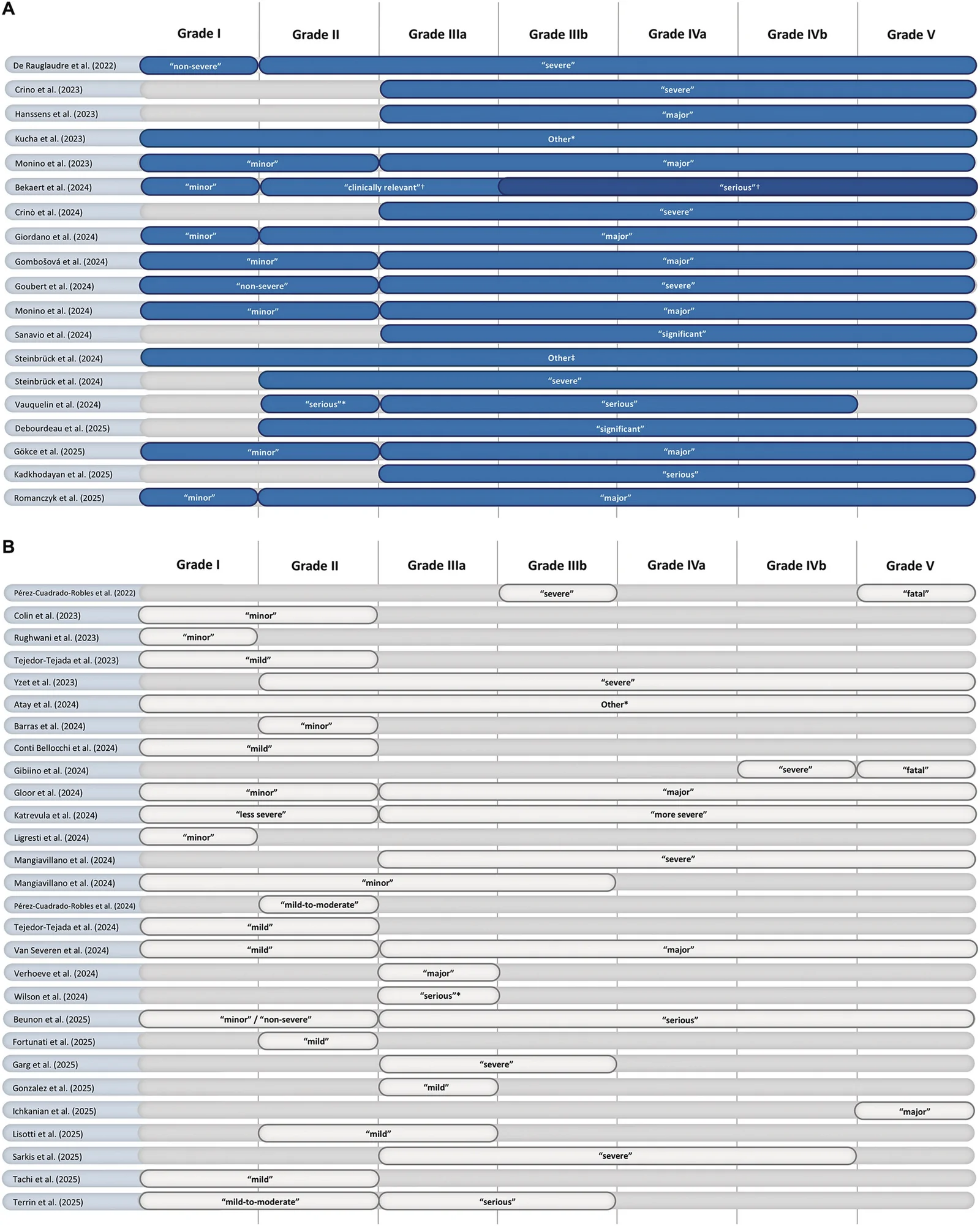
( **A** ) The use of subjective terminology in relation to adverse event (AE) severity as determined using the AGREE classification: articles for which used terminology was specified within the methods section. *Bleeding, infection, perforation, peritonitis, buried bumper syndrome, and procedure-related death (within 24 h of the procedure) were considered major AEs. Peristomal leakage, tube dislodgement, and other AEs manageable within an outpatient setting were considered minor AEs; †All AEs with a severity of grade II or higher were defined as “clinically relevant” and AEs with a severity grade IIIb or higher were defined as “serious”; ‡Intra- or postprocedural perforation and postendoscopic bleeding were considered major AEs. Other AEs were considered minor AEs. Note: a detailed reference list is available in **Appendix S2** (available in the online version). ( **B** ) The use of subjective terminology in relation to AE severity as determined using the AGREE classification: articles for which used terminology was not specified within the methods section. *Major bleeding and perforation were considered major AEs. Minor bleeding, lumen-apposing metal stent (LAMS) blockage, procedure-related infection and LAMS-migration were considered minor AEs. Note: a detailed reference list is available in **Appendix S2** (available in the online version).

Findings were used to develop a checklist to guide clinicians in more standardized definition and reporting of AEs in scientific reports and publications ( Table 2 ).

**Table 2:** Checklist for standardized reporting of adverse events in scientific reports and publications using the AGREE classification.

**Methods section**
Describe the used definition for AEs In case of deviation from the use of the standard AGREE definition*, provide rationale Describe how AEs were identified and where AEs were registered or recorded
Describe the timeframe (follow-up period) during which AEs were monitored If the timeframe exceeds 30 post-procedural days, as is standard using the AGREE classification, provide rationale
If applicable, describe the different groups of patients, practitioners, procedures, or institutions for which AEs were evaluated
Describe all formal statistical analyses conducted using AE data
**Results section**
Report the incidence, type, and severity of all AEs In case no AEs occurred, state so Specifically report the AE rate for the 30-days postprocedural timeframe If the timeframe during which AEs were monitored exceeds 30 postprocedural days, report results for the period after the 30-days postprocedural period separately Provide severity grades for all AEs (grade I-V) In case of grade III and IV AEs, report grade IIIa and IIIb and grade IVa and IVb AEs separately (ensure inclusion of suffixes) If clinically relevant, report AEs for all groups of patients, practitioners, procedures, or institutions separately Avoid the use of subjective terminology related to severity or timing of (the onset of) AEs
Consider reporting events that did not meet the criteria for an AE according to the AGREE classification criteria, but may be relevant to document for other purposes, as “incidents”†
Consider providing additional textual information (aside from severity grades) for any AE that is considered of substantial clinical relevance, as well as for unexpected or unusual AEs.

Abbreviation: AEs, adverse events.

*“A negative outcome for the patient that prevents completion of a planned procedure or causes any deviation from the standard postprocedural course. Events during the procedure that did not change the standard postprocedural course are not regarded as adverse events. Adverse events should be recorded in an intention-to-scope manner, thus for all scheduled procedures.”

†See recommendations provided with Table 1 .

## Discussion

The results of this study suggest a gradual annual increase in the use of the AGREE classification for classification of GI endoscopy-related AEs. Moreover, the literature reviewed indicates that the AGREE classification facilitates intradisciplinary comparisons across endoscopic treatment modalities, patient populations, endoscopists, and institutions, 14 15 16 17 18 19 20 21 22 23 24 25 26 27 28 29 30 31 32 33 34 35 36 37 38 39 40 41 42 43 44 45 46 as well as interdisciplinary comparisons encompassing endoscopic, radiological, and surgical approaches. 49 50 51 52 The latter is particularly relevant given the substantial increase in therapeutic (interventional) endoscopic procedures that have replaced surgical interventions over the past decade, 86 a trend that is expected to continue over the coming years. Complementing these findings, evidence suggests that AE severities assessed using the AGREE classification correlate significantly with those assessed using other established classifications, 5 53 54 and that the AGREE classification may achieve superior inter-observer agreement compared to the ASGE classification. 54

Collectively, the findings of this study suggest that the AGREE classification holds promise to become a widely used tool for standardized assessment of GI endoscopy-related AEs. It must be acknowledged, however, that the citation-based methodology employed in this review principally captures the academic visibility of, and scholarly interest in, the AGREE classification. Direct evidence involving the adoption and impact of the classification in routine clinical practice, endorsement by institutions and professional societies, or real-world user experience were not obtained through the current study. Despite the lack of such insights, this study may offer valuable insights into the current position of the AGREE classification within the field of GI endoscopy, and inform strategies to optimize the identification, assessment, and reporting of AEs in routine clinical practice.

Primarily, to further contextualize our study findings, the citation trajectory of the original AGREE publication 5 can be compared to that of the longer-established ASGE classification. 6 Data from the SCOPUS database indicate annual citation counts for the AGREE classification of 10, 37, and 66 in 2022, 2023, and 2024, respectively. While these figures remain lower than those recorded for the ASGE classification over the same period (ranging between 232 and 254), they are comparable to the citation counts observed for the ASGE classification during its early years (increasing from 4 to 38 between 2010 and 2013). These metrics suggest that the academic visibility of the AGREE classification is increasing at a pace broadly similar to that of the ASGE classification during its initial years. Meanwhile, these metrics should be interpreted with some caution considering the likely lower research output and less uniform registry of endoscopy-related AEs at the time of the introduction of the ASGE classification.

Second, AE grading using the AGREE classification was illustrated to not always be straightforward. While it should be emphasized that our intention was not to retrospectively critique individual assessments or assign blame for potential misclassifications, through the performed review of identified scenarios and the additional recommendations provided we aimed to offer additional practical guidance for appraisal of specific (categories of) more challenging AE scenarios ( Table 1 ). Particularly, the recommendation to report events without significant sequelae for patients as “incidents,” rather than AEs, could assist in mitigating the overclassification of AEs that contributed to the majority (77%) of observed misclassifications.

Third, inconsistencies in identification and reporting of AEs were observed. Most importantly, we observed that the timeframe during which AEs were monitored is frequently not specified. Besides, considerable heterogeneity in adopted timeframes was found across studies that did delineate a follow-up duration ( **Table S2** , available in the online version). This is of clinical relevance as with different follow-up durations (ranging from two weeks up to over multiple years across identified studies; **Table S2** , available in the online version), the incidence of AEs may vary considerably. Besides, as time progresses, distinguishing events that are directly attributable to the initial endoscopic procedure (i.e., procedural risk) from those more likely caused by factors such as natural disease course may become increasingly challenging. Therefore, to improve consistency in defining and reporting AEs, the original AGREE recommendations should be reiterated: all AEs occurring within 30 days of an endoscopic procedure should be recorded, irrespective of the likelihood of a link between the event and the endoscopic procedure. The likelihood of an AE being related to the procedure should be determined through subsequent discussions (e.g., during regular team meetings), thereby minimizing the risk of subjective interpretation. 5 Consistent reporting of AEs with an onset within the 30-days postprocedural timeframe across all studies, complemented by separate reporting of longer-term follow-up outcomes if relevant, would enhance the comparability of results.

Discrepancies were also observed in the definitions and classifications used for AE severity grading, which may substantially affect the generalizability of study findings. 40 Furthermore, some articles did not specify AE severities according to AGREE classification grades, or did so inconsistently. 33 69 70 71 72 73 74 75 76 77 Although comprehensive textual descriptions of the AE scenarios were mostly available, thereby allowing readers to infer severity, the absence of specific grades could impose subjective interpretation. Finally, we noted frequent use of subjective terminology related to both the severity and timing of AEs. While such language may facilitate more fluent reporting, a wide variety of terms were used to describe AEs of similar severity ( Fig. 4A,B ). Similarly, used terminology related to the timing of AEs was observed to yield significantly different implications across studies ( **Table S2** , available in the online version). Such discrepancies impose an additional risk for (erroneous) subjective interpretation, thereby potentially impairing the robustness of AE comparisons across studies.

To address observed inconsistencies in the definition and reporting of AEs, a checklist for AE reporting was developed ( Table 2 ). This checklist incorporates targeted recommendations addressing the definition- and reporting-related issues identified in this study, alongside more general guidance adapted from existing AE reporting checklists. 10 11 12 13 With the checklist, we aim to support physicians to ensure that published reports: (a) clearly specify the AE definition used; (b) adequately define the timeframe during which AEs were monitored; (c) ensure complete AE reporting; and (d) minimize reliance on subjective or nonstandardized terminology in relation to AE severity and timing (of their onset).

The main strength of this study relates to the fact that the literature search, data extraction, and initial review of (potential) AE scenarios were independently conducted by two authors, thereby enhancing the robustness of the reported data. Moreover, the identified challenges related to accurate AE grading using the AGREE classification, as well as the observed variability in the definition and reporting of AEs, were addressed through additional recommendations rather than adjustments to the classification itself – an approach that preserves continuity for existing users and is likely to facilitate broader acceptance and more consistent application of the AGREE classification going forward.

This study also yields various limitations. First, it relied on a citation-based analysis rather than a systematic literature search. Although this approach was deliberate, given that most databases do not support direct full-text searches, it may have excluded studies applying the AGREE classification without citing the index publication. Nevertheless, the number of such studies is likely small, as reference screening did not result in the identification of any additional eligible articles. Second, no formal consensus process was employed to develop the additional recommendations for grading the specific (challenging) AE scenarios included in Table 1 . Consequently, these recommendations reflect expert opinion from the authors of the original AGREE publication rather than a consensus-derived extension of the classification. The absence of a formal consensus procedure may also have introduced some degree of bias in evaluating the accuracy of AE classifications using the AGREE classification, considering that expert opinion rather than formal consensus represented the reference standard for AE severity grades.

Future research involving the AGREE classification is warranted across several domains. Most importantly, dedicated studies are needed to evaluate the extent of its implementation in routine practice settings, its endorsement by health care institutions and professional societies, and the associated real-world user experience. Repeating the international survey on standard practices for monitoring and evaluating GI endoscopy-related AEs 5 could provide valuable insights in this regard. Additionally, as of January 2024, the AGREE classification has replaced the ASGE classification as the standard in the Dutch Registry of Complications in Endoscopy 87 ; in due course, this may facilitate the first nationwide assessment of the implications of adopting the AGREE classification as the (national) standard for AE evaluation. Moreover, prior studies have suggested a significant correlation between severity grades as assessed using both the AGREE and ASGE classifications. 5 53 54 Besides, greater inter-observer agreement using the AGREE classification has been reported. 54 However, as this evidence only involves a limited number of studies, further independent validation of the correlation between AGREE-based AE assessments with assessment using other classifications is needed. Finally, future studies should investigate the extent to which the additional recommendations proposed in this study improve the consistency of AE grading, definition, and reporting.

In conclusion, the gradual annual increase in citations of the AGREE classification suggests growing awareness and scholarly interest, while the illustrated feasibility to facilitate a wide range of intra- and interdisciplinary AE comparisons underscores its potential to serve as a valuable tool for AE assessment in GI endoscopy. Citation trends alone, however, do not constitute evidence of broad clinical validity and feasibility, and further real-world data on the implementation and institutional adoption of the classification, as well as user experience involving the classification, are needed. Besides, inconsistencies in AE definition and reporting remain common, and AE grading using the AGREE classification is not always straightforward. We hope that the additional recommendations provided in this review will contribute to further minimization of inconsistencies in AE identification, grading, and reporting, thereby improving opportunities for robust GI endoscopy quality evaluation and optimization.

## Supplementary Material

Ergänzendes Material Supplementary Material
