# Supplementary material for: The Classification for Adverse Events in GastRointEstinal Endoscopy (AGREE): Three-Year Evaluation and Additional Recommendations
Source: Endosc Int Open. 2026 Oct 13;14:a29656729. doi: 10.1055/a-2965-6729 (PMC13662677; doi:10.1055/a-2965-6729)
Supplement: Supplementary file 1 [file 10-1055-a-2965-6729_29813251.pdf]

SUPPLEMENTARY MATERIALS

The Classification for Adverse Events in GastRointEstinal Endoscopy (AGREE): Three-Year Evaluation and Additional Recommendations

Querijn N.E. van Bokhorst; Hessel Bierma; Karlijn J. Nass; Evelien Dekker; Manon van der Vlugt; Paul Fockens

TABLE OF CONTENTS

Table S1 Characteristics of original research articles using the AGREE classification for classification of (potential) adverse events related to gastrointestinal endoscopy 2

Table S2 Evaluation of adverse event reporting practices within original research articles using the AGREE classification for grading (potential) adverse events related to gastrointestinal endoscopy 10

Table S3 (Potential) adverse event scenarios as reported within original research articles using the AGREE classification 19

Table S4 Adverse event definition and period during which adverse events should be monitored according to different classification systems 32

Table S5 Adverse event definition and period during which adverse events should be monitored according to different classifications 33

Appendix S1 Detailed description and evaluation of complex (adverse) event scenarios 34

Appendix S2 Reference list for Figures 4A-B 39

Endosc Int Open | DOI 10.1055/a-2965-6729 | © The Author(s).

Table S1 Characteristics of original research articles using the AGREE classification for classification of (potential) adverse events related to gastrointestinal endoscopy

Year	Author	Countries*	Study design†	Single- vs. multi-center	Endoscopy: category	Endoscopy: topic(s)
**2022**	Pérez-Cuadrado-Robles et al. [1]	FRA	Prospective‡, observational	Single-center	Upper GI, intervention	EUS-guided gastroenterostomy
**2022**	De Rauglaudre et al. [2]	FRA	Retrospective, observational	Multi-center	Upper GI, diagnostic	Endoscopic resection of early-stage cancer
**2023**	Aelvoet et al. [3]	DNK, ESP, GBR, ITA, NLD, POL	Prospective‡, observational	Multi-center	Upper GI, intervention	Cold snare polypectomy of duodenal adenomas
**2023**	Borrelli de Andreis et al. [4]	ITA	Retrospective, observational	Single-center	HPB, intervention	EUS-guided radiofrequency ablation
**2023**	Colin et al. [5]	BEL	Retrospective, observational	Single-center	Enteroscopy, diagnostic	Motorized spiral enteroscopy
**2023**	Crinò et al. [6]	BEL, CHE, DNK, FRA, IND, IRN, ISR, ITA, NOR	Retrospective, observational	Multi-center	HPB, intervention	EUS-guided radiofrequency ablation
**2023**	Crispino et al. [7]	ITA	Prospective‡, observational	Single-center	Multiple, diagnostic and intervention	Validation of the AGREE classification
**2023**	Falt et al. [8]	CHE	Prospective, observational	Single-center	Enteroscopy, diagnostic	Motorized spiral enteroscopy
**2023**	Geyl et al. [9]	FRA	Prospective, observational	Single-center	HPB, intervention	EUS-guided biliary drainage
**2023**	Ghazi et al. [10]	USA	Retrospective, observational	Single-center	HPB, intervention	Biliary stenting
**2023**	Hanssens et al. [11]	BEL	Retrospective, observational	Single-center	HPB, intervention	EUS-guided and PTC-guided biliary cannulation
**2023**	Hayat et al. [12]	USA	Retrospective, observational	Multi-center	Multiple, intervention	ESD
**2023**	Jaroenlapnopparat et al. [13]	USA	Retrospective, observational	Single-center	Upper GI, intervention	Transoral outlet reduction
**2023**	Khan et al. [14]	USA	Prospective, interventional	Single-center	Upper GI, intervention	POEM

Endosc Int Open | DOI 10.1055/a-2965-6729 | © The Author(s).

**2023**	Kucha et al. [15]	POL	Retrospective, observational	Single-center	Upper GI, intervention	Percutaneous endoscopic gastrostomy
**2023**	Liu et al. [16]	CHN	Prospective, interventional	Single-center	HPB, intervention	Biliary cannulation
**2023**	Mangiavillano et al. [17]	ITA	Retrospective, observational	Multi-center	Upper GI, intervention	EUS-guided gastroenterostomy
**2023**	Momblan et al. [18]	ESP	Prospective, observational	Multi-center	Upper GI, intervention	Endoscopic vacuum therapy
**2023**	Monino et al. [19]	BEL, FRA	Retrospective, observational	Multi-center	Upper GI, intervention	EUS-guided gastroenterostomy
**2023**	Mutignani et al. [20]	ITA	Prospective‡, observational	Single-center	Enteroscopy, intervention	Endoscopic entero-enteral bypass
**2023**	Nakamura et al. [21]	JPN	Retrospective, observational	Single-center	Upper GI, intervention	EUS-guided hepaticogastrostomy
**2023**	Napoleon et al. [22]	FRA	Retrospective, observational	Multi-center	HPB, intervention	EUS-guided radiofrequency ablation
**2023**	Pattynama et al. [23]	NLD	Prospective, interventional	Single-center	Upper GI, intervention	Endoscopic vacuum therapy and an intraluminal stenting
**2023**	Pérez-Cuadrado-Robles et al. [24]	FRA	Retrospective, observational	Single-center	Upper GI, intervention	ERCP following EUS-guided gastro-gastrostomy
**2022**	Rughwani et al. [25]	IND	Prospective, RCT	Single-center	Enteroscopy, diagnostic	Motorized spiral and single-balloon enteroscopy
**2023**	Stefanovic et al. [26]	BEL	Retrospective, observational	Single-center	HPB, intervention	LAMS-related adverse events
**2023**	Tejedor-Tejada et al. [27]	ESP	Retrospective, observational	Single-center	Upper GI, intervention	Esophageal stenting
**2023**	Tiebie et al. [28]	NLD	Prospective, RCT	Multi-center	Upper GI, intervention	Esophageal food bolus impaction
**2023**	Weiss et al. [29]	FRA	Retrospective, observational	Single-center	Upper GI, intervention	Endoscopic radiofrequency ablation
**2023**	Wessels et al. [30]	NLD	Retrospective, observational	Single-center	Upper GI, intervention	POEM

Endosc Int Open | DOI 10.1055/a-2965-6729 | © The Author(s).

**2023**	Yzet et al. [31]	FRA	Prospective‡, observational	Multi-center	Lower GI, intervention	ESD and EFTR
**2024**	Abad et al. [32]	ESP	Prospective, RCT	Multi-center	Upper GI, intervention	Endoscopic sleeve gastroplasty
**2024**	Albers et al. [33]	CZE, DEU, ITA, USA	Retrospective, observational	Multi-center	Upper GI, intervention	POEM
**2024**	Atay et al. [34]	TUR	Prospective‡, observational	Single-center	HPB, intervention	Endoscopic and percutaneous management of WON
**2024**	Barras et al. [35]	BEL	Retrospective, observational	Single-center	Upper GI, intervention	POEM
**2024**	Bekaert et al. [36]	BEL	Retrospective, observational	Single-center	Multiple, intervention	ESD
**2024**	Castro et al. [37]	BRA	Retrospective, observational	Single-center	HPB, diagnostic	EUS-guided biopsy
**2024**	Chon et al. [38]	KOR	Retrospective, observational	Single-center	HPB, intervention	EUS-guided gallbladder drainage
**2024**	Conti Bellocchi et al. [39]	ITA	Retrospective, observational	Multi-center	Upper GI, intervention	EUS-guided gastroenterostomy and enteral stenting
**2024**	Crinò et al. [40]	ITA	Retrospective, observational	Single-center	HPB, intervention	EUS-guided fine-needle biopsy and ERCP
**2024**	Dell’Anna et al. [41]	ITA	Prospective‡, observational	Single-center	Upper GI, intervention	Endoscopic management of post-esophagectomy delayed gastric conduit emptying
**2024**	Drews et al. [42]	DEU	Retrospective, observational	Single-center	HPB, intervention	Endoscopic biliary drainage
**2024**	Fritzsche et al. [43]	NLD	Prospective, interventional	Single-center	HPB, intervention	EUS-guided choledochoduodenostomy
**2024**	Fritzsche et al. [44]	NLD	Prospective, interventional	Single-center	HPB, intervention	EUS-guided choledochoduodenostomy
**2024**	Gibiino et al. [45]	ITA	Retrospective, observational	Multi-center	Lower GI, intervention	EFTR
**2024**	Giordano et al. [46]	ESP	Prospective, observational	Multi-center	Enteroscopy, diagnostic and intervention	Motorized spiral enteroscopy

Endosc Int Open | DOI 10.1055/a-2965-6729 | © The Author(s).

**2024**	Gloor et al. [47]	CHE	Retrospective, observational	Single-center	HPB, intervention	Antegrade ballon dilatation during ERCP
**2024**	Gombošová et al. [48]	SVN	Retrospective, observational	Single-center	Upper GI, intervention	Adverse events related to parenteral nutrition and medication administration
**2024**	Goubert et al. [49]	BEL	Retrospective, observational	Single-center	HPB, intervention	ERCP-related adverse events
**2024**	Jürgensen et al. [50]	DEU	Retrospective, observational	Single-center	HPB, intervention	EUS-guided ablation
**2024**	Katrevula et al. [51]	IND	Prospective, observational	Single-center	Multiple, diagnostic and intervention	Audit of endoscopy-related adverse events
**2024**	Khalaf et al. [52]	USA	Retrospective, observational	Single-center	Multiple, intervention	ESD
**2024**	Koduri et al. [53]	IND	Prospective, RCT	Single-center	HPB, intervention	EUS-guided drainage of WON
**2024**	Ligresti et al. [54]	ITA	Retrospective, observational	Multi-center	HPB, intervention	Endoscopic necrosectomy
**2024**	Lisotti et al. [55]	ITA	Retrospective, observational	Multi-center	HPB, diagnostic	EUS-guided fine-needle biopsy
**2024**	Mangiavillano et al. [56]	CRI, ITA, KOR, SAU, USA	Prospective, interventional	Multi-center	HPB, intervention	EUS-guided gallbladder drainage
**2024**	Mangiavillano et al. [57]	BEL, CHE, CRI, ECU, IND, ITA, NOR, SAU, USA	Retrospective, observational	Multi-center	HPB, intervention	LAMS in patients with surgically altered anatomy
**2024**	Masciangelo et al. [58]	ITA	Prospective, interventional	Single-center	HPB, intervention	ERCP in patients with moderate-to-severe cholangitis
**2024**	Mesureur et al. [59]	AUS, BEL, CHE, GBR, FRA, ITA, NLD, SWE	Prospective‡, observational	Multi-center	Upper GI, intervention	ESD
**2024**	Meulen et al. [60]	NLD	Prospective, RCT	Multi-center	Lower GI, intervention	EMR
**2024**	Mohammed et al. [61]	USA	Prospective, observational	Multi-center	Lower GI, intervention	Endoscopic resection of colorectal polyps
**2024**	Monino et al. [62]	BEL	Retrospective, observational	Single-center	HPB, intervention	Endoscopic management of biliary and pancreatic pathologies in Roux-en-Y gastric bypass patients

Endosc Int Open | DOI 10.1055/a-2965-6729 | © The Author(s).

**2024**	Moon et al. [63]	KOR	Prospective, RCT	Multi-center	HPB, intervention	EUS-guided drainage of infected WON
**2024**	Moura et al. [64]	BRA	Retrospective, observational	Single-center	Lower GI, diagnostic	Quality indicators for screening colonoscopy
**2024**	Nimri et al. [65]	USA	Retrospective, observational	Multi-center	HPB, intervention	Adverse events associated with transmural use of LAMS
**2024**	Pérez-Cuadrado-Robles et al. [66]	BEL, DEU, FRA, GBR, ITA	Retrospective, observational	Multi-center	HPB, intervention	PTC
**2024**	Rancatore et al. [67]	ITA	Retrospective, observational	Single-center	HPB, diagnostic	EUS-guided fine needle biopsy
**2024**	Rodriguez de Santiago et al. [68]	AUS, BEL, CHE, DEU, ESP, FRA, GBR, NLD, POL, PRT	Prospective‡, observational	Multi-center	Upper GI, intervention	ESD
**2024**	Roskovicova et al. [69]	SVK	Retrospective, observational	Single-center	HPB, intervention	Risk factors for ERCP-related adverse events
**2024**	Ruiz-Manriquez et al. [70]	MEX	Retrospective, observational	Single-center	HPB, intervention	Averse events in patients with surgically altered gastrointestinal anatomy undergoing ERCP
**2024**	Sadeghi et al. [71]	IRN	Prospective, RCT	Single-center	HPB, intervention	Needle-knife fistulotomy and standard transpapillary technique for cannulation of long-size papillas
**2024**	Sadeghi et al. [72]	IRN	Prospective‡, observational	Single-center	HPB, intervention	Needle-knife fistulotomy for deep biliary cannulation
**2024**	Sanavio et al. [73]	FRA	Retrospective, observational	Multi-center	Upper GI, intervention	POEM
**2024**	Steinbrück et al. [74]	DEU	Prospective, RCT	Multi-center	Lower GI, intervention	Endoscopic resection of colorectal polyps
**2024**	Steinbrück et al. [75]	DEU	Retrospective, observational	Multi-center	Upper GI, intervention	Endoscopic treatment of Zenker's diverticulum
**2024**	Stigliano et al. [76]	ITA	Retrospective, observational	Multi-center	HPB, intervention	Timing of LAMS removal in pancreatic fluid collections
**2024**	Tejedor-Tejada et al. [77]	ESP	Retrospective, observational	Single-center	Upper GI, intervention	Secondary stenting for the management of occluded biliary stents
**2024**	van Severen et al. [78]	BEL, FRA	Retrospective, observational	Multi-center	Upper GI, intervention	POEM

Endosc Int Open | DOI 10.1055/a-2965-6729 | © The Author(s).

**2024**	Vargas-Madrigal et al. [79]	CRI, FRA, HKG, IND	Prospective, interventional	Multi-center	HPB, intervention	EUS-guided hepaticogastrostomy
**2024**	Vauquelin et al. [80]	FRA	Retrospective, observational	Multi-center	Upper GI, intervention	POEM
**2024**	Velasquez-Rodriguez et al. [81]	ESP	Retrospective, observational	Multi-center	HPB, intervention	EUS-guided simple puncture-aspiration
**2024**	Verhoeve et al. [82]	NLD	Retrospective, observational	Single-center	Upper GI, diagnostic	EUS-guided keyhole biopsies
**2024**	Wang et al. [83]	CHN	Retrospective, observational	Single-center	Upper GI, intervention	Endoscopic resection of nonampullary duodenal neuroendocrine tumors
**2024**	Wessels et al. [84]	NLD	Retrospective, observational	Single-center	Upper GI, intervention	POEM
**2024**	Wilson et al. [85]	USA, CAN	Retrospective, observational	Multi-center	Upper GI, intervention	Cold snare EMR of nonampullary duodenal adenomas
**2024**	Yang et al. [86]	USA	Retrospective, observational	Multi-center	Multiple, intervention	ESD
**2024**	Yang et al. [87]	USA	Prospective, observational	Single-center	Upper GI, intervention	POEM and pyloric muscle biopsies
**2024**	Yang et al. [88]	USA	Prospective, interventional	Multi-center	Upper GI, intervention	ESD
**2025**	Balducci et al. [89]	CHE, FRA	Retrospective, observational	Multi-center	HPB, intervention	EUS-guided hepaticojejunostomy
**2025**	Beunon et al. [90]	BEL, CHE, FRA	Retrospective, observational	Multi-center	HPB, intervention	EUS-guided choledochoduodenostomy
**2025**	Bronswijk et al. [91]	BEL	Prospective‡, interventional	Multi-center	HPB, intervention	EUS-directed ERCP
**2025**	Dahel et al. [92]	FRA	Retrospective, observational	Single-center	HPB, intervention	Intrabiliary radiofrequency ablation
**2025**	Debourdeau et al. [93]	FRA	Retrospective, observational	Multi-center	HPB, intervention	EUS-guided choledochoduodenostomy and EUS-guided gallbladder drainage
**2025**	Fortunati et al. [94]	BEL	Prospective‡, observational	Single-center	HPB, intervention	ERCP in patients with total and partial gastrectomy

Endosc Int Open | DOI 10.1055/a-2965-6729 | © The Author(s).

**2025**	Garg et al. [95]	ESP, USA	Retrospective, observational	Multi-center	HPB, intervention	EUS-guided gallbladder drainage
**2025**	Gökce et al. [96]	BEL	Prospective‡, observational	Single-center	Multiple, intervention	LAMS for creation of anastomoses
**2025**	Gonzalez et al. [97]	FRA	Retrospective, observational	Single-center	Upper GI, intervention	EUS-guided gastroenterostomy
**2025**	Ichkhanian et al. [98]	ESP, GBR, USA	Retrospective, observational	Multi-center	HPB, intervention	EUS-guided drainage of intra-abdominal abscesses
**2025**	Kadkhodayan et al. [99]	USA	Prospective, observational	Single-center	HPB, intervention	EUS-guided ERCP in patients with Roux-en-Y gastric bypass
**2025**	Lee et al. [100]	USA	Prospective‡, observational	Single-center	Upper GI, intervention	EUS-guided gastroenterostomy
**2025**	Lisotti et al. [101]	FRA, ITA, ISR	Prospective‡, observational	Multi-center	Lower GI, intervention	EUS-guided pelvic abscess drainage
**2025**	Martinez-Moreno et al. [102]	ESP	Prospective, observational	Single-center	HPB, diagnostic	EUS-guided portal pressure gradient measurement
**2025**	Mega et al. [103]	BRA	Prospective‡, observational	Single-center	Upper GI, intervention	Endoscopic vacuum therapy
**2025**	Neri et al. [104]	FRA, ITA	Retrospective, observational	Multi-center	Lower GI, intervention	EUS-guided entero-colostomy
**2025**	Ogura et al. [105]	JPN	Retrospective, observational	Single-center	HPB, intervention	EUS-guided pancreaticogastrostomy
**2025**	Pollack et al. [106]	USA	Retrospective, observational	Single-center	Multiple, intervention	LAMS for benign GI strictures
**2025**	Romanczyk et al. [107]	POL	Retrospective, observational	Multi-center	Upper GI, intervention	Endoscopic ablation
**2025**	Salame et al. [108]	CZE, JOR, USA	Prospective‡, observational	Multi-center	Upper GI, intervention	POEM
**2025**	Sarkis et al. [109]	USA	Retrospective, observational	Single-center	Upper-GI, intervention	Needle-knife septomy and POEM
**2025**	Shiomi et al. [110]	JPN	Retrospective, observational	Multi-center	HPB, intervention	Endoscopic drainage of WON

Endosc Int Open | DOI 10.1055/a-2965-6729 | © The Author(s).

**2025**	Tachi et al. [111]	JPN	Retrospective, observational	Single-center	HPB, intervention	EUS-guided hepaticogastrostomy
**2025**	Terrin et al. [112]	FRA	Retrospective, observational	Single-center	Multiple, intervention	EUS-guided drainage of post-operative fluid collections
**2025**	Wannhoff et al. [113]	CHE, DEU, USA	Retrospective, observational	Multi-center	Upper GI, intervention	Endoscopic ultrasound-guided gastroenterostomy

*Country abbreviations as displayed within this column are based on the ISO 3166-1 alpha-3 codes from the Internation Organization for Standardization: AUS, Australia; BEL, Belgium; BRA, Brazil; CAN, Canada; CHE, Switzerland; CHN, China; CRI, Costa Rica; DEU, Germany; DNK, Denmark; ECU, Ecuador; ESP, Spain; FRA, France; GBR, United Kingdom of Great Britain and Northern Ireland; HKG, Hong Kong; IND, India; IRN, Iran; ISR, Israel; ITA, Italy; JOR, Jordan; JPN, Japan; KOR, Republic of Korea; MEX, Mexico; NLD, the Netherlands; NOR, Norway; POL, Poland; PRT, Portugal; SAU, Saudi Arabia; SVK, Slovakia; SVN, Slovenia; SWE, Sweden; TUR, Turkey; USA, United States of America; †Cohort studies, case-control studies, cross-sectional studies and case series were all defined as observational studies; ‡Retrospective analysis of (a) prospectively collected data(set).

GI, gastrointestinal; EUS, endoscopic ultrasound; HPB, hepato-pancreato-biliary; PTC, percutaneous transhepatic cholangiography; ESD, endoscopic submucosal dissection; POEM, peroral endoscopic myotomy; ERCP, endoscopic retrograde cholangiopancreatography; RCT, randomized clinical trial; LAMS, lumen-apposing metal stent; EFTR, endoscopic full-thickness resection; WON, walled-off necrosis; EMR, endoscopic mucosal resection.

Endosc Int Open | DOI 10.1055/a-2965-6729 | © The Author(s).

Table S2 Evaluation of adverse event reporting practices within original research articles using the AGREE classification for grading (potential) adverse events related to gastrointestinal endoscopy

Year	Author	Used classification(s)	Differentiation Grades III and IV 'a' and 'b'*	Use of subjective terminology related to grades of the AGREE classification	Definition time-related cut-offs for reporting adverse events	Use of subjective terminology related to timing of adverse events†
**2022**	Pérez-Cuadrado-Robles et al. [1]	AGREE	Yes	Single grade IIIb and grade V AE referred to as "severe" and "fatal"	No	No
**2022**	De Rauglaudre et al. [2]	AGREE	Yes	Grade I: "non-severe"; Grade $\geq$II: "severe"‡	Within 30 days‡	No
**2023**	Aelvoet et al. [3]	AGREE	*NA*	No	Within 30 days‡	No
**2023**	Borrelli de Andreis et al. [4]	AGREE (with ASGE definition)	*NA*	No	No	<48 hours (during hospitalization): "early"
**2023**	Colin et al. [5]	AGREE (with ASGE definition), Clavien-Dindo	*NA*	Grade I-II referred to as "minor"	No	No
**2023**	Crinò et al. [6]	AGREE, ASGE	No§	Grade $\geq$III: "severe"‡	No	No
**2023**	Crispino et al. [7]	AGREE, ASGE	Yes	No	No	No
**2023**	Falt et al. [8]	AGREE (with other definition)	Yes	No	Within 30 days	No
**2023**	Geyl et al. [9]	AGREE, ASGE	Yes	No	No	During hospitalization: "early"‡
**2023**	Ghazi et al. [10]	AGREE	Yes	No	No	No
**2023**	Hanssens et al. [11]	AGREE	Yes	Grade $\geq$III: "major"‡	No	No
**2023**	Hayat et al. [12]	AGREE	*NA*	No	No	No
**2023**	Jaroenlapnopparat et al. [13]	AGREE (referred to as ASGE classification)	No¶	No	No	No
**2023**	Khan et al. [14]	AGREE	*NA*	No	No	No

Endosc Int Open | DOI 10.1055/a-2965-6729 | © The Author(s).

**2023**	Kucha et al. [15]	AGREE	Yes	Specific subtypes of AEs referred to as "major" (bleeding, infection, perforation, peritonitis, buried bumper syndrome, death)‡	From the date of the procedure (PEG placement) until PEG removal, adverse event occurrence, death, or end of study (whichever occurred first)‡	No
**2023**	Liu et al. [16]	AGREE	*NA*	No	No	"Late AEs" (without definition)
**2023**	Mangiavillano et al. [17]	AGREE	*NA*	No	AE evaluation at 7, 30 and 60 days, and then monthly thereafter‡	During the procedure: "intraprocedural"; <u><</u>24 hours: "early"; >24 hours: "late"‡
**2023**	Momblan et al. [18]	AGREE (with other definition)	Yes	No	AE evaluation at 1, 3 and 6 months after conclusion of therapy‡	No
**2023**	Monino et al. [19]	AGREE	Yes	Grade I-II: "minor"; Grade <u>></u>III: "major"‡	During the follow-up period (median 5 months)‡	No
**2023**	Mutignani et al. [20]	AGREE	No§	No	No	<u><</u>1 week: "early"; >1 week: "late"‡
**2023**	Nakamura et al. [21]	AGREE	*NA*	No	No	No
**2023**	Napoleon et al. [22]	AGREE	Yes	No	Within one month‡	No
**2023**	Pattynama et al. [23]	AGREE	*NA*	No	Within 30 days‡	No
**2023**	Pérez-Cuadrado-Robles et al. [24]	AGREE (with ESGE definition)	Yes	No	No	No
**2023**	Rughwani et al. [25]	AGREE	*NA*	Grade I referred to as "minor"	Within 30 days‡	No
**2023**	Stefanovic et al. [26]	AGREE (referred to as ASGE classification)	Yes	No	No	No
**2023**	Tejedor-Tejada et al. [27]	AGREE (with other definition)	*NA*	Grade I-II referred to as "mild"	Until patient death‡	<u><</u>24 hours: "early"; >24 hours: "delayed"‡
**2023**	Tiebie et al. [28]	AGREE	*NA*	No	Within one week‡	No

Endosc Int Open | DOI 10.1055/a-2965-6729 | © The Author(s).

**2023**	Weiss et al. [29]	AGREE	No§	No	During the follow-up period; median 29 months‡	No
**2023**	Wessels et al. [30]	AGREE	Yes	No	Within 30 days‡	No
**2023**	Yzet et al. [31]	AGREE	Yes	Grade II-V referred to as "severe"	Within one month (for bleedings specifically)‡	No
**2024**	Abad et al. [32]	AGREE	*NA*	No	No	No
**2024**	Albers et al. [33]	AGREE, ASGE	Yes	No	No	No
**2024**	Atay et al. [34]	AGREE	*NA*	Use of "minor" and "major" terminology without corresponding AGREE grades	No	No
**2024**	Barras et al. [35]	AGREE (and according to ASGE recommendations)	*NA*	Grade II referred to as "minor"	No	No
**2024**	Bekaert et al. [36]	AGREE	Yes	Grade I: "minor";<br>Grade ≥II: "clinically relevant";<br>Grade ≥IIIb: "serious"‡	No	"Late strictures" (without definition)
**2024**	Castro et al. [37]	AGREE (with other definition)	*NA*	No	During follow-up of at least one year or until the final outcome of the evolution‡	No
**2024**	Chon et al. [38]	AGREE	No§	No	No	During the procedure: "intraprocedural";<br>≤14 days: "early";<br>>14 days: "late"‡
**2024**	Conti Bellocchi et al. [39]	AGREE (with ASGE definition)	*NA*	Grade I-II referred to as "mild"	During the follow-up; median 91 and 92 months‡	During the procedure: "intraprocedural";<br>≤7 days: "early";<br>>7 days: "late"‡
**2024**	Crinò et al. [40]	AGREE (with ASGE definition of 'incidents')	Yes	Grade ≥III: severe‡	No	No
**2024**	Dell'Anna et al. [41]	AGREE	*NA*	No	During the study period (median follow-up of 374 and 230 days in involved groups, respectively)	No
**2024**	Drews et al. [42]	AGREE	Yes	No	No	No

**2024**	Fritzsche et al. [43]	AGREE	Yes	No	Within 30 days‡	No
**2024**	Fritzsche et al. [44]	AGREE	Yes	No	Within 30 days‡	No
**2024**	Gibiino et al. [45]	AGREE	Yes	Grade IV-V referred to as "severe" or "fatal	During follow-up of at least 3 months‡	During the procedure: "intraprocedural";<br>After the procedure had been completed: "post-procedural"
**2024**	Giordano et al. [46]	AGREE	Yes	Grade I: "minor";<br>Grade ≥II: "major"‡	Within 14 days‡	During the procedure: "periprocedural";<br>≤24 hours: "early";<br>>24 hours "delayed"
**2024**	Gloor et al. [47]	AGREE, Clavien-Dindo	Yes	Grade I-II referred to as "minor";<br>Grade ≥III referred to as "major"	No	No
**2024**	Gombošová et al. [48]	AGREE	Yes	Grade I-II: "minor";<br>Grade ≥III: "major"‡	No	≤7 days: "early periprocedural";<br>>7 days: "late"‡
**2024**	Goubert et al. [49]	AGREE	No§	Grade I-II: "non-severe";<br>Grade ≥III: "severe"‡	Within 30 days (for mortality specifically)	No
**2024**	Jürgensen et al. [50]	AGREE	*NA*	No	During follow-up period (variable for each patient)‡	No
**2024**	Katrevula et al. [51]	AGREE	Yes	Grade I-II referred to as "less severe";<br>Grade III-V referred to as "more severe"	Within 30 days‡	No
**2024**	Khalaf et al. [52]	AGREE (with ASGE definition)	Yes	No	No	No
**2024**	Koduri et al. [53]	AGREE (with other definition)	*NA*	No	During the study period (up until 6 months)‡	No
**2024**	Ligresti et al. [54]	AGREE	*NA*	Grade I referred to as "minor"	Within one month‡	During the procedure: "intraprocedural";<br>After the procedure: "delayed"
**2024**	Lisotti et al. [55]	AGREE (possibly with ASGE definition)	*NA*	No	No	No
**2024**	Mangiavillano et al. [56]	AGREE (with ASGE definition)	Yes	Grade ≥III: "severe"	No	During the procedure: "intraprocedural";<br>≤48 hours: "early";<br>>48 hours: "late"‡

Endosc Int Open | DOI 10.1055/a-2965-6729 | © The Author(s).

**2024**	Mangiavillano et al. [57]	AGREE	Yes	Stated that only “minor” AEs occurred (with AEs up to grade IIIb reported)	No	<u><</u>48 hours: “early”; >48 hours: “late”‡
**2024**	Masciangelo et al. [58]	AGREE (with ESGE definition)	Yes	No	During follow-up of 3 months‡	No
**2024**	Mesureur et al. [59]	AGREE	Yes	No	No	No
**2024**	Meulen et al. [60]	AGREE	Yes	No	No	During the procedure: “intraprocedural”; <u><</u>24 hours: “early” (bleedings); >24 hours: “delayed” (bleedings)‡
**2024**	Mohammed et al. [61]	AGREE	*NA*	No	Within 30 days‡	During the procedure: “intraprocedural; After the procedure: “delayed” (bleedings)‡
**2024**	Monino et al. [62]	AGREE	Yes	Grade I-II: “minor”; Grade <u>></u>III: “major”‡	During the study follow-up period (not further defined)‡	No
**2024**	Moon et al. [63]	AGREE	Yes	No	No	No
**2024**	Moura et al. [64]	AGREE	*NA*	No	No	No
**2024**	Nimri et al. [65]	AGREE, ASGE	Yes	No	No	<u><</u>48 hours: “early”; >48 hours: “late”‡
**2024**	Pérez-Cuadrado-Robles et al. [66]	AGREE	*NA*	Grade II referred to as “mild-to-moderate”	No	No
**2024**	Rancatore et al. [67]	AGREE	*NA*	No	No	No
**2024**	Rodriguez de Santiago et al. [68]	AGREE	No**	No	Within 30 days (for bleedings specifically)‡	During the procedure: “intraprocedural”; After the procedure: “delayed”‡
**2024**	Roskovicova et al. [69]	AGREE	Yes	No	No	Acute pancreatitis, bleeding, cholangitis, infection, and intestinal perforation: “early”
**2024**	Ruiz-Manriquez et al. [70]	AGREE	Yes	No	Within the first month‡	No
**2024**	Sadeghi et al. [71]	AGREE	*NA*	No	Within 30 days‡	During hospitalization: “immediate”; After discharge: “delayed”
**2024**	Sadeghi et al. [72]	AGREE	No§	No	Within 30 days‡	During hospitalization: “immediate”; After discharge: “delayed”

Endosc Int Open | DOI 10.1055/a-2965-6729 | © The Author(s).

**2024**	Sanavio et al. [73]	AGREE	Yes	Grade ≥III: “significant”‡	No	No
**2024**	Steinbrück et al. [74]	AGREE	Yes	Specific AEs defined as “major” (subtypes of perforations and bleedings)‡	During follow-up (4 weeks to 6 months)‡	No
**2024**	Steinbrück et al. [75]	AGREE	Yes	Grade II-V: “severe”‡	No	No
**2024**	Stigliano et al. [76]	AGREE (with other definition)	Yes	No	No	≤4 weeks: “early”; >4 weeks: “late”‡
**2024**	Tejedor-Tejada et al. [77]	AGREE	*NA*	Grade I-II referred to as “mild”	No	No
**2024**	van Severen et al. [78]	AGREE	No¶	Grade I-II referred to as “mild”; Grade III-V referred to as “major”	No	No
**2024**	Vargas-Madrigal et al. [79]	AGREE	*NA*	No	No	No
**2024**	Vauquelin et al. [80]	AGREE	Yes	Grade II with prolonged hospitalization (>3 days), grade III and grade IV: “serious”‡	No	No
**2024**	Velasquez-Rodriguez et al. [81]	AGREE (referred to as ASGE classification)	Yes	No	No	No
**2024**	Verhoeve et al. [82]	AGREE	Yes	A single grade IIIa AE referred to as “major”	Within the first month‡	No
**2024**	Wang et al. [83]	AGREE	Yes	No	Within two weeks (for bleedings specifically)‡	No
**2024**	Wessels et al. [84]	AGREE	Yes	No	Within 30 days‡	No
**2024**	Wilson et al. [85]	AGREE	*NA*	Single grade IIIa AE referred to as “serious” (specific AGREE grade not provided, but textual description of the AE available)	Within 30 days‡	During the procedure: “immediate”; After the procedure: “delayed”‡
**2024**	Yang et al. [86]	AGREE	*NA*	No	Within 30 days (for bleedings specifically)‡	No

Endosc Int Open | DOI 10.1055/a-2965-6729 | © The Author(s).

**2024**	Yang et al. [87]	AGREE	*NA*	No	No	No
**2024**	Yang et al. [88]	AGREE	No§	No	At least 30 days or till onset of symptoms	No
**2025**	Balducci et al. [89]	AGREE	Yes	No	Within the first month‡	No
**2025**	Beunon et al. [90]	AGREE	Yes	Grade I-II: "minor";<br>Grade ≥III: "serious" (AGREE grades not specifically provided within article)	Within 30 days‡	≤30 days: "early" or "short-term"
**2025**	Bronswijk et al. [91]	AGREE	*NA*	No	No	No
**2025**	Dahel et al. [92]	AGREE	Yes	No	Only AEs occurring ≤30 days were graded using the AGREE classification, events occurring >30 days were reported without grading‡	≤30 days: "morbidity";<br>>30 days: "late complications"‡
**2025**	Debourdeau et al. [93]	AGREE	Yes	Grade >II: significant‡	No	<24 hours: "early",<br>>24 hours: "delayed"‡
**2025**	Fortunati et al. [94]	AGREE	No**	Grade II referred to as "mild"	Within 30 days (for mortality specifically)	No
**2025**	Garg et al. [95]	AGREE	Yes	Three grade IIIa/b AEs referred to as "severe"	No	≤48 hours: "early" (bleedings);<br>>48 hours: "late" (bleedings)‡
**2025**	Gökce et al. [96]	AGREE	Yes	Grade I-II: "minor";<br>Grade ≥III: "major"‡	No	No
**2025**	Gonzalez et al. [97]	AGREE	No§	Grade IIIa AE referred to as "mild"	No	No
**2025**	Ichkhanian et al. [98]	AGREE	Yes	Single grade V AE referred to as "major"	No	≤24 hours: "immediate",<br>>24 hours: "late"
**2025**	Kadkhodayan et al. [99]	AGREE	*NA*	Grade ≥III: "serious"‡	At least 3 months‡	No
**2025**	Lee et al. [100]	AGREE<br>(referred to as ASGE)	Yes	No	Within 30 days‡	No
**2025**	Lisotti et al. [101]	AGREE (with EUS ASGE lexicon definition)	Yes	A single grade II and IIIa AE referred to as "mild"	No	During the procedure: "procedural";<br>After the procedure: "late"

Endosc Int Open | DOI 10.1055/a-2965-6729 | © The Author(s).

**2025**	Martinez-Moreno et al. [102]	AGREE	Yes	No	At least 30 days, additional review of medical records for delayed adverse events (undefined time range)‡	No
**2025**	Mega et al. [103]	AGREE	*NA*	No	No (but primary study outcome concerned no rebleeding ≤30 days)	No
**2025**	Neri et al. [104]	AGREE	*NA*	No	No	No
**2025**	Ogura et al. [105]	AGREE (referred to as ASGE)	*NA*	No	No	No
**2025**	Pollack et al. [106]	AGREE (referred to as ASGE)	No§	No	No	No
**2025**	Romanczyk et al. [107]	AGREE	*NA*	Grade I: “minor”; Grade ≥II: “major”‡	No	No
**2025**	Salame et al. [108]	AGREE	*NA*	No	No	During the procedure: “intraprocedural”; ≤48 hours: “immediate”; <15 days: “delayed”‡
**2025**	Sarkis et al. [109]	AGREE	No§	Grade III and IV AEs referred to as “severe”	Within 30 days‡	No
**2025**	Shiomi et al. [110]	AGREE, ASGE	No	No	Within study-follow or until death‡	No
**2025**	Tachi et al. [111]	AGREE	*NA*	Grade I-II referred to as “mild”	No	No
**2025**	Terrin et al. [112]	AGREE (with ASGE definition)	Yes	Grade I-II referred to as “mild-to-moderate”; Grade IIIa/b referred to as “serious”	Until at least 6 months or death	During procedure or within ≤48 hours: “periprocedural”; >48 hours: “delayed”‡
**2025**	Wannhoff et al. [113]	AGREE	Yes	No	No	No

*For studies for which *NA* is reported, this indicates that either no grade III adverse event(s) occurred or the specific AGREE severity grades of the adverse event(s) were not provided; †Adverse events classified only as “intraprocedural” or “post-procedural” were not included; ‡Defined explicitly in the study’s methods section; §AGREE grades reported but incompletely specified (e.g., “grade III” without distinction between IIIa or IIIb) - textual descriptions of consequences or treatments are available; ¶ No AGREE grades reported, but adverse event descriptions include consequences and/or required treatments; ** Inconsistent differentiation of severity grades throughout the article (e.g., IIIa vs. IIIb distinguished, but not IVa vs. IVb).
AE, adverse event; NA, not applicable; ASGE, American Society for Gastrointestinal Endoscopy; PEG, percutaneous endoscopic gastrostomy; ESGE, European Society for Gastrointestinal Endoscopy.

Endosc Int Open | DOI 10.1055/a-2965-6729 | © The Author(s).

Table S3 (Potential) adverse event scenarios as reported within original research articles using the AGREE classification								
**Year**	**Author**	**Number of cases**	**Type of AE (summary of original description)**	**Consequence and/or required treatment (summary of original description)**	**Time between endoscopy and AE**	**AGREE grade authors**	**AGREE grade review**	**Remark**
**2022**	Pérez-Cuadrado-Robles et al. [1]	1	Colonic perforation due to delayed stent migration	Death of the patient	12 days	IV	V	Death of the patient, therefore this AE should be considered a grade V AE
		2	Delayed colonic perforation	Surgical intervention	0 days	IIIb	IIIb	*NA*
**2022**	De Rauglaudre et al. [2]	2	Bleeding	Transfusion and prolonged hospital stay (undefined duration)	Unspecified, but ≤30 days*	II	II	*NA*
		1	Gastric perforation	Endoscopic intervention and ICU admission	Unspecified, but ≤30 days*	IVa	IVa or IVb	Based on the case description it is uncertain whether the patient suffered from single- or multiple organ failure, therefore this AE could either concern a grade IVa or IVb AE
		1	Aspiration pneumonia due to aspiration during anesthesia	Multiple organ failure and death of the patients 10 days following the procedure	Unspecified, but ≤30 days*	V	V	*NA*
**2023**	Borrelli de Andreis et al. [4]	2	Mild post-procedural abdominal pain without signs of pancreatitis or perforation	Single dose of non-opioid analgesics	Unspecified	I	I	*NA*
**2023**	Colin et al. [5]	13	Superficial mucosal lacerations in the upper esophagus	No clinical consequences, no treatment required	Unspecified	I	No AE	See evaluation and recommendation as provided in Table 1 (Scenario 1)
		1	Superficial mucosal lacerations in the jejunum	No clinical consequences, no treatment required	Unspecified	I	No AE	See evaluation and recommendation as provided in Table 1 (Scenario 1)
		2	Post-procedural abdominal pain, most likely related to intestinal perforation	CT scan, antibiotics	Unspecified	II	II	*NA*
		1	Biliary leak	Antibiotics	Unspecified	II	II	*NA*
**2023**	Crinò et al. [6]	1	Spleen hematoma	Hospital admission >24 hours	Unspecified	II	II	*NA*
		4	Abdominal pain	Conservative management	Unspecified	I	I	*NA*
		1	Abdominal pain	Antibiotics	Unspecified	II	II	*NA*
		1	Diabetes	Medical treatment (not further defined)	Unspecified	II	II	*NA*

Endosc Int Open | DOI 10.1055/a-2965-6729 | © The Author(s).

2023	Falt et al. [8]	1	(Mild) acute pancreatitis	Hospital stay prolongation by 2 days	Unspecified, but ≤30 days*	II	II	*NA*
		1	Intussusception during endoscope withdrawal	Resolved by parallel insertion of a pediatric colonoscope, with one episode of post-procedural hematochezia, hospital stay prolongation by 1 day	0 days (intraprocedural)	IIIa	I or II	See evaluation and recommendation as provided in Table 1 (Scenario 3)
		3	Mucosal tear of the esophagus	No clinical consequences, no treatment required	Unspecified, but ≤30 days*	No AE	No AE	*NA*
2023	Geyl et al. [9]	1	Distal flange misdeployment in the peritoneal cavity	Percutaneous biliary drainage	0 days	IIIa	IIIa	*NA*
		2	Distal flange misdeployment in the peritoneal cavity	Immediate successful new EUS-guided choledochoduodenostomy with another LAMS	0 days (intraprocedural)	I	No AE	See evaluation and recommendation as provided in Table 1 (Scenario 2)
		1	Distal flange misdeployment in the peritoneal cavity	EUS-guided gallbladder drainage	0 days	IIIa	IIIa	*NA*
2023	Hayat et al. [12]	3	Post-procedural abdominal pain	Analgesics and hospital admission (<24 hours)	0 days	I	I	*NA*
		1	Post-procedural abdominal pain	Not requiring analgesics or hospital admission	0 days	No AE	No AE	*NA*
2023	Mutignani et al. [20]	4	Spontaneous stent displacement	Insertion of another stent through the stent	Unspecified, but >1 week	IIIa	IIIa	*NA*
2023	Pérez-Cuadrado-Robles et al. [24]	1	Anastomotic dehiscence during routine follow-up ERCP 5 days after gastrojejunal anastomosis	Surgery	5 days	IIIb	IIIb	Note: AE should be documented as an AE related to the second procedure (ERCP)
2023	Rughwani et al. [25]	5	Deep esophageal tears, duodenal tear and/or colonic mucosal tear	No clinical consequences, no treatment required	0 days (intraprocedural)	I	No AE	See evaluation and recommendation as provided in Table 1 (Scenario 1)
2023	Stefanovic et al. [26]	1	Melena	Repeat endoscopy and CT angiogram were performed, but no bleeding source was identified – treatment with fluid resuscitation	Unspecified	IIIa	IIIa	*NA*
		4	Gastrointestinal bleeding	Endoscopic intervention	Unspecified	IIIa	IIIa	*NA*
		1	Gastrointestinal bleeding	Endoscopic intervention and blood transfusion	Unspecified	IIIa	IIIa	*NA*
		2	LAMS dysfunction due to tumor overgrowth	Endoscopy to remove or replace LAMS	Unspecified	IIIa	No AE or IIIa	See evaluation and recommendation as provided in Table 1 (Scenario 6)

Endosc Int Open | DOI 10.1055/a-2965-6729 | © The Author(s).

		1	LAMS-related enterocolonic fistula	Endoscopy to remove or replace LAMS	Unspecified	IIIa	IIIa	*NA*
		1	LAMS-related air and fluid leaks	Endoscopy to remove or replace LAMS	Unspecified	IIIa	IIIa	*NA*
		1	LAMS-related ascending cholangitis	Endoscopy to remove or replace LAMS	Unspecified	IIIa	IIIa	*NA*
		1	Deployment of the distal flange of the LAMS into rectal muscle tissue	LAMS removed, conservative management due to patient's complex surgical history and comorbidities	Unspecified	IIIa	IIIa	*NA*
		1	LAMS dislocation during ERCP 8 days following gastro-gastrostomy, resulting in perforation	Surgery	Unspecified	IIIb	IIIb	Note: AE should be documented as an AE related to the second procedure (ERCP)
**2023**	Tiebie et al. [28]	6	Mucosal lesions	No clinical consequences, no treatment required	Unspecified, but ≤1 week	No AE	No AE	*NA*
		1	Vomiting during endoscopy	No clinical consequences, no treatment required	0 days (intraprocedural)	No AE	No AE	*NA*
		1	Minimal bleeding	No clinical consequences, no treatment required	Unspecified, but ≤1 week	No AE	No AE	*NA*
**2023**	Weiss et al. [29]	1	Gastrointestinal bleeding	Repeat endoscopy and hospital admission (undefined duration)	7 days	III	IIIa	Considering that repeat endoscopy was required, this AE concerns a grade IIIa AE
		14	Esophageal strictures	Hydrostatic dilatation	Unspecified	III	IIIa	Considering that repeat endoscopy was required, these AEs concern grade IIIa AEs
**2023**	Wessels et al. [30]	1	Mucosal injury during the procedure	Closure with four extra hemoclips	0 days (intraprocedural)	No AE	No AE	*NA*
		1	Post-procedural retrosternal pain	Readmission (undefined duration), treatment with non-opioid analgesics	Undefined, but ≤30 days*	II	I or II	The case description provides no insights into the duration of the hospital admission: based on duration of the hospital stay (≤24 hours or >24 hours) this concerns either a grade I or II AE
		1	Post-procedural retrosternal pain and partial dehiscence of the mucosal incision after POEM	Readmission (undefined duration), placement of a duodenal feeding tube for 25 days, healing of the dehiscence under conservative management	Undefined, but ≤30 days*	IIIa	IIIa	*NA*
**2024**	Albers et al. [33]	1	Covered perforation	Endoscopic intervention and prolonged hospital stay (<10 days)	Unspecified	IIIa	IIIa	*NA*

Endosc Int Open | DOI 10.1055/a-2965-6729 | © The Author(s).

		1	Pneumothorax	Chest tube drainage and prolonged hospital stay (<10 days)	Unspecified	IIIa	IIIa	*NA*
		1	Mucosal defect and dehiscence of entry	Endoscopic intervention	Unspecified	IIIa	IIIa	*NA*
		1	Unstable angina pectoris	Cardiological care, prolonged hospital stay <10 days	Unspecified	II	II	*NA*
		1	Nosocomial pneumonia	Antibiotics, CT scan, prolonged hospital stay <10 days	Unspecified	II	II	*NA*
		1	Dyspnea, chest pain, increased CRP	Antibiotics, CT scan, prolonged hospital stay <10 days	Unspecified	II	II	*NA*
		1	Mucosal lesion (clipped), fever, chest pain, increased CRP	Antibiotics, prolonged hospital stay <10 days	Unspecified	II	II	*NA*
		1	Contralateral mucosal tear (clipped) and mucosal lesion	Standard and repeated endoscopic control without intervention, antibiotics, prolonged stay <10 days	Unspecified	II	II	*NA*
		1	Fever, increased CRP	Antibiotics, prolonged hospital stay ≤3 days	Unspecified	II	II	*NA*
		2	Mucosal lesions (clipped)	No clinical consequences, no treatment required	Unspecified	No AE	No AE	*NA*
		1	Oxygen insufflation leading to emphysema and pneumothorax	X-ray, spontaneous recovery without intervention	Unspecified	No AE	I	Presuming that the performed X-ray concerned an unscheduled X-ray (not part of routine clinical care), this case should be considered a grade I AE
		2	Distal attachment lost in the submucosal tunnel	No clinical consequences, no treatment required	Unspecified	No AE	No AE	*NA*
**2024**	Barras et al. [35]	1	Post-procedural pain	Analgesics and prolonged hospital stay	Unspecified	II	I or II	The case description provides no insights into the duration of the hospital admission: based on duration of the hospital stay (≤24 hours or >24 hours) this concerns either a grade I or II AE
		2	Asymptomatic pneumothorax	Spontaneous recovery without intervention, but requiring 48 hours of in-hospital monitoring	Unspecified	II	II	*NA*
**2024**	Chon et al. [38]	1	Intraprocedural duodenal perforation	Surgery	0 days (intraprocedural)	III	IIIb	Considering that surgery was required, this AE concerns a grade IIIb AE
		2	Intraprocedural stent migration into the gallbladder	Placement of a covered stent along the previously placed guidewire	0 days (intraprocedural)	III	No AE	See evaluation and recommendation as provided in Table 1 (Scenario 2)

Endosc Int Open | DOI 10.1055/a-2965-6729 | © The Author(s).

		1	Stent obstruction due to food, resulting in acute cholecystitis	Endoscopic balloon sweeping and placement of double-pigtail stents	Unspecified, but ≤14 days	III	IIIa	Considering that repeat endoscopy was required, this AE concerns a grade IIIa AE
		1	Acute cholangitis	Endoscopic intervention	Unspecified, but >14 days	III	IIIa	Considering that repeat endoscopy was required, this AE concerns a grade IIIa AE
		1	Recurrent acute cholecystitis due to stent obstruction	Endoscopic balloon sweeping and placement of double-pigtail stents	Unspecified, but >14 days	III	IIIa	Considering that repeat endoscopy was required, this AE concerns a grade IIIa AE
**2024**	Crinò et al. [40]	9	Bleeding after sphincterotomy	Endoscopic intervention		IIIa	IIIa	*NA*
		6	Cholangitis	Endoscopic intervention		IIIa	IIIa	*NA*
		5	Stent dysfunction/migration	Endoscopic intervention		IIIa	IIIa	*NA*
		5	Septic shock secondary to cholangitis	ICU admission (undefined duration)		IV	IVa or IVb	Based on the case description it is uncertain whether the patient suffered from single- or multiple organ failure, therefore this AE could either concern a grade IVa or IVb AE
**2024**	Giordano et al. [46]	4	Difficult and prolonged withdrawal in the mid-proximal esophagus during motorized antegrade enteroscopy	No intervention, but 24-36 hours of in-hospital observation		II	II	*NA*
		5	Melena after reintroduction of acenocoumarol 48 hours after enteroscopy during which a 20 mm polyp was resected	Observation for 48 hours, no intervention		II	II	*NA*
		6	Post-procedural abdominal pain due to acute jejunal ischemia (potentially related to treatment of a 10 mm angiodysplasia in the jejunum)	Surgery		IIIb	IIIb	*NA*
**2024**	Goubert et al. [49]	1	Post-procedural rectal blood loss due to papillotomy-related bleeding	Endoscopic intervention (adrenaline injection and coagulation)		III	IIIa	Considering that repeat endoscopy was required, this AE concerns a grade IIIa AE
		1	Post-procedural melena due to papillotomy-related bleeding	Repeat endoscopy, no intervention		III	IIIa	Considering that repeat endoscopy was required, this AE concerns a grade IIIa AE
		3	Post-procedural anemia due to papillotomy-related bleeding	Endoscopic intervention (adrenaline injection and coagulation)		III	IIIa	Considering that repeat endoscopy was required, these AEs concerns grade IIIa AEs
		2	Post-procedural melena due to papillotomy-related bleeding	Endoscopic intervention (adrenaline injection and coagulation)		III	IIIa	Considering that repeat endoscopy was required, these AEs concerns grade IIIa AEs
		1	Post-procedural melena due to papillotomy-related bleeding	Endoscopic intervention (placement of covered metallic stent)		III	IIIa	Considering that repeat endoscopy was required, this AE concerns a grade IIIa AE

Endosc Int Open | DOI 10.1055/a-2965-6729 | © The Author(s).

		1	Post-procedural anemia due to papillotomy-related bleeding	Endoscopic intervention (adrenaline injection)		III	IIIa	Considering that repeat endoscopy was required, this AE concerns a grade IIIa AE
		1	Post-procedural rectal blood loss	Repeat endoscopy, no intervention (no active bleeding visualized)		III	IIIa	Considering that repeat endoscopy was required, this AE concerns a grade IIIa AE
		1	Post-procedural melena, possibly due to papillotomy-related bleeding	Endoscopic intervention (adrenaline injection of the papilla due to presumed papillotomy-related bleeding)		III	IIIa	Considering that repeat endoscopy was required, this AE concerns a grade IIIa AE
		1	Post-procedural melena after stent placement for a malignant biliary obstruction	Repeat endoscopy, no active bleeding visualized (possible bleeding of the tumor)		III	IIIa	Considering that repeat endoscopy was required, this AE concerns a grade IIIa AE
		1	Post-procedural melena due to bleeding between two plastic biliary stents	Endoscopic intervention (removal of stent, replacement with metallic stent)		III	IIIa	Considering that repeat endoscopy was required, this AE concerns a grade IIIa AE
		1	Post-procedural melena due to post-papillotomy bleeding	Radiological intervention (embolization)		III	IIIa	Considering that repeat endoscopy was required, this AE concerns a grade IIIa AE
		2	Post-procedural melena and hemorrhagic shock due to post-papillotomy bleeding	ICU admission (undefined duration), endoscopic intervention		IV	IVa or IVb	Based on the case description it is uncertain whether the patient suffered from single- or multiple organ failure, therefore this AE could either concern a grade IVa or IVb AE
		1	Hematemesis and hemorrhagic shock due to pseudoaneurysm	ICU admission (undefined duration), radiological intervention (embolization)		IV	IVa or IVb	Based on the case description it is uncertain whether the patient suffered from single- or multiple organ failure, therefore this AE could either concern a grade IVa or IVb AE
		2	Cholangiosepsis with shock	Multiple organ failure and death		V	V	*NA*
		1	Ventricular fibrillation with circulatory arrest during ERCP	Unsuccessful resuscitation, death of the patient		V	V	*NA*
		1	Hemorrhagic shock due to bleeding after unsuccessful cannulation and a false passage during ERCP	Unsuccessful embolization and surgical intervention but hemostasis could not be established, death of the patient		V	V	*NA*
		1	Duodenal perforation after ERCP and EUS-guided rendezvous	Supportive care due to patient comorbidities, death of the patient		V	V	*NA*
		1	Post-procedural melena and hemorrhagic shock, likely due to papillotomy-related bleeding	ICU admission, repeat endoscopy, death of the patient		V	V	*NA*
**2024**	Koduri et al. [53]	1-2	Intraprocedural bleeding at the puncture site	Self-limiting, not requiring intervention	Unspecified	No AE	No AE	*NA*

Endosc Int Open | DOI 10.1055/a-2965-6729 | © The Author(s).

		1	Significant brisk bleeding	Crossover from plastic stent to a biflanged metal stent and blood transfusion	Unspecified	IIIa	II	See evaluation and recommendation as provided in Table 1 (Scenario 8)
**2024**	Ligresti et al. [54]	18	Intraprocedural bleeding	No drop in hemoglobin levels, modification of procedure course or need for further surgical/radiologic intervention	Unspecified, but ≤1 month	I	No AE	See evaluation and recommendation as provided in Table 1 (Scenario 1)
		2	LAMS displacement	Intraprocedural management by placement of a new LAMS, no clinical consequences	0 days (intraprocedural)	I	No AE	See evaluation and recommendation as provided in Table 1 (Scenario 2)
**2024**	Mangiavillano et al. [56]	1	Over bleeding 24 hours after the procedure	Radiological intervention (embolization)	1 day	IIIa	IIIa	*NA*
		1	LAMS occlusion due to food impaction	Endoscopic intervention (removal of the food)	13 days	IIIa	IIIa	See evaluation and recommendation as provided in Table 1 (Scenario 5)
		1	Recurrent jaundice due to tumoral cystic duct occlusion	Radiological intervention (percutaneous transhepatic biliary drainage)	6 weeks	IIIa	No AE	See evaluation and recommendation as provided in Table 1 (Scenario 6)
		1	Recurrent jaundice due to tumoral cystic duct occlusion	Endoscopic intervention (hepaticogastrostomy)	8 weeks	IIIa	No AE	See evaluation and recommendation as provided in Table 1 (Scenario 6)
**2024**	Masciangelo et al. [58]	1	Stent migration causing delayed duodenal bleeding after ERCP	Endoscopic intervention (through-the-scope hemoclip)	Unspecified	IIIa	IIIa	*NA*
		1	Self-limiting bleeding of the duodenal bulb, close to the proximal flange of a LAMS deployed for EUS-guided gallbladder drainage	Endoscopic intervention (placement of a double pigtail soft plastic stent)	Unspecified	IIIa	IIIa	*NA*
**2024**	Mesureur et al. [59]	1	Muscular tear	Treated with stent placement during the procedure	0 days (intraprocedural)	IIIa	No AE	See evaluation and recommendation as provided in Table 1 (Scenario 2)
		2	Transmural perforation	Treated with hemoclips during the procedure	0 days (intraprocedural)	No AE	No AE	*NA*
		5	Muscular tear(s)	Two out of five treated with hemoclips during the procedure	0 days (intraprocedural)	No AE	No AE	*NA*
		6	Esophageal strictures	Balloon dilatations (2-9 sessions)	Unspecified	IIIa	IIIa	*NA*
**2024**	Nimri et al. [65]	1	Distal displacement of LAMS into the cyst (detected during repeat routine endoscopy)	Redeployment of a new LAMS and removal of the old LAMS	30 days	I	No AE	See evaluation and recommendation as provided in Table 1 (Scenario 8)
		1	Displacement of the LAMS into the excluded stomach (detected during repeat routine endoscopy)	Fixation of the LAMS with endoscopic suturing	2 days	I	No AE	See evaluation and recommendation as provided in Table 1 (Scenario 8)

Endosc Int Open | DOI 10.1055/a-2965-6729 | © The Author(s).

		1	Displacement of the LAMS into the stomach (detected during repeat routine endoscopy)	None, conservative treatment	45 days	I	No AE	Event with an onset >30 days after the initial procedure
		1	Mild non-bleeding gastric mucosal erosion adjacent to the LAMS (detected during repeat routine endoscopy)	None, conservative treatment	38 days	I	No AE	Event with an onset >30 days after the initial procedure
		1	Mild non-bleeding gastric mucosal erosion adjacent to the LAMS (detected during repeat routine endoscopy)	None, conservative treatment	23 days	I	No AE	See evaluation and recommendation as provided in Table 1 (Scenario 8)
		1	Blood clots inside the LAMS without signs of active bleeding (detected during unscheduled repeat endoscopy)	None, conservative management	2 days	IIIa	IIIa	*NA*
		1	Blood clots inside the LAMS without signs of active bleeding (detected during routine repeat endoscopy)	None, conservative management	27 days	I	No AE	See evaluation and recommendation as provided in Table 1 (Scenario 8)
		1	Sepsis after transgastric LAMS deployment for management of acute cholecystitis	ICU admission	0 days	IVa	IVa	Presuming the patient was admitted to the ICU for single organ failure, grade IVa is considered correct
		1	Pulseless electrical activity with return of spontaneous circulation due to a pulmonary embolism	ICU admission	0 days	IVa	IVa	Presuming the patient was admitted to the ICU for single organ failure, grade IVa is considered correct
**2024**	Sadeghi et al. [72]	1	Perforation after fistulotomy for deep biliary cannulation	Placement of a fully covered self-expandable metal stent during the procedure	0 days (intraprocedural)	III	No AE	Presuming recovery without sequalae and no alterations to expected post-procedural course: see evaluation and recommendation as provided in Table 1 (Scenario 2)
**2024**	Stigliano et al. [76]	2	Bleeding	Prolongation of the hospital stay and blood transfusion	Unspecified, but mostly ≤4 weeks	II	II	*NA*
		5	Bleeding	Radiological intervention	Unspecified, but mostly ≤4 weeks	IIIa	IIa	*NA*
**2024**	van Severen et al. [78]	1	Bilateral capnothorax, capnomediastinum and capnoperitoneum due to accidental room air insufflation; pulmonary embolism	ICU admission, needle exsufflation and placement of two surgical chest drains; anticoagulation treatment	0 days (intraprocedural)	IV	IVa or IVb	Based on the case description it is uncertain whether the patient suffered from single- or multiple organ failure, therefore this AE could either concern a grade IVa or IVb AE
		2	Capnothorax resulting in cardiorespiratory arrest	Cardiopulmonary resuscitation and needle exsufflation of the capnothorax, ICU admission	0 days (intraprocedural)	IV	IVa or IVb	Based on the case description it is uncertain whether the patient suffered from single- or

Endosc Int Open | DOI 10.1055/a-2965-6729 | © The Author(s).

								multiple organ failure, therefore this AE could either concern a grade IVa or IVb AE
**2024**	Velasquez-Rodriguez et al. [81]	1	Puncture-related bleeding	Radiological intervention	Unspecified	IIIa	IIIa	*NA*
		2	Puncture-related abdominal pain	Hospital admission (3 days) and antibiotics	Unspecified	II	II	*NA*
**2024**	Verhoeve et al. [82]	1	Hematemesis related to a post-biopsy bleeding	Endoscopic intervention (placement of an additional hemoclip at the biopsy site)	0 days	IIIa	IIIa	*NA*
**2024**	Wang et al. [83]	1	Delayed perforation	Endoscopic debridement, fistula drainage, enteral nutrition and antibiotics	Unspecified (hospital stay 13 days)	IIIa	IIIa	*NA*
		2	Delayed perforation	Endoscopic debridement, fistula drainage, enteral nutrition and antibiotics	Unspecified (hospital stay 53 days)	IIIa	IIIa	*NA*
		3	Delayed bleeding	Endoscopic intervention	Unspecified (hospital stay 8 days)	IIIa	IIIa	*NA*
		4	Delayed bleeding	Endoscopic intervention	Unspecified (hospital stay 15 days)	IIIa	IIIa	*NA*
**2024**	Wessels et al. [84]	1	Retrosternal pain	Opioids for pain control and prolonged hospital stay >24 hours	1 day	II	II	*NA*
		1	Esophageal leakage	Repeat endoscopy with placement of extra clips, antibiotics, duodenal tube feeding, prolonged hospital stay >24 hours	1 day	IIIa	IIIa	*NA*
		1	Esophageal leakage	Two repeat endoscopies with placement of extra clips, duodenal tube feeding, prolonged hospital stay >24 hours	1 day	IIIa	IIIa	*NA*
		1	Esophageal leakage	Opioids for pain control, prolonged hospital stay <24 hours	1 day	I	I	*NA*
		1	Intraprocedural bleeding	Prolonged observation (<24 hours), no blood transfusion required	1 day	I	I	*NA*
		1	Retrosternal pain, pneumoperitoneum	Opioids, CT scan, antibiotics for two days (until perforation ruled out by upper endoscopy), readmission for 48 hours	4 days	IIIa	IIIa	*NA*

Endosc Int Open | DOI 10.1055/a-2965-6729 | © The Author(s).

1	Melena due to ulcer in the cardia where previously mucosal injury was clipped during POEM	Diagnostic repeat endoscopy, conservative treatment	21 days	IIIa	IIIa	*NA*
1	Low blood pressure post-procedure	No treatment, prolonged hospital stay <24 hours (prolonged observation)	0 days	I	I	*NA*
1	Retrosternal pain, no abnormalities on imaging	Non-opioids for pain control, chest X-ray, prolonged hospital stay <24 hours	0 days	I	I	*NA*
1	Pneumoperitoneum, pneumomediastinum, subcutaneous emphysema and pneumothorax after inadvertently using room air instead of carbon dioxide during POEM	Opioids for pain control, chest X-ray and CT, prolonged hospital stay >24 hours	0 days	II	II	*NA*
1	Retrosternal pain, no abnormalities on imaging	Opioids for pain control, CT scan, visit to emergency department without readmission	1 day	I	I	*NA*
1	Pneumonia	Chest X-ray, antibiotics for 5 days	1 day	II	II	*NA*
1	Intraprocedural bleeding and difficult closure of mucosal incision	Nasogastric tube until esophageal leakage was ruled out, CT scan, prolonged hospital stay <24 hours (prolonged observation)	0 days	I	I	*NA*
1	Retrosternal pain, pneumoperitoneum	Non-opioids for pain control, CT scan, prolonged hospital stay <24 hours	1 day	I	I	*NA*
1	Low-grade fever and retrosternal pain	Readmission for observation (1 night and day), CT, non-opioids for pain control	2 days	I	I	*NA*
1	Abdominal pain and respiratory insufficiency due to pneumonia with pleural effusion	ICU admission, CT, high flow oxygen, antibiotics, pleural drainage	3 days	IVa	IVa	*NA*
1	Retrosternal pain and dehiscence of the mucosal incision	Readmission for 1 night, endoscopy with placement of feeding tube, non-opioids for pain control, duodenal tube feeding for 25 days	5 days	IIIa	IIIa	*NA*
1	Retrosternal pain, pneumoperitoneum	Visit to the emergency department without need for readmission, non-opioids for pain control	3 days	I	I	*NA*
1	Retrosternal pain after POEM, no evident signs for esophageal leakage	Non-opioids for pain control, prolonged hospital stay <24 hours	1 day	I	I	*NA*

Endosc Int Open | DOI 10.1055/a-2965-6729 | © The Author(s).

**2024**	Yang et al. [88]	1	Post-procedural bleeding from an exposed submucosal vessel at the resection bed	Endoscopic intervention	0 days	III	IIIa	*NA*
		2	Post-procedural bleeding, no active bleeding identified at time of repeat endoscopy	Repeat endoscopy without intervention, discharge within 24 hours	0 days	I	IIIa	Although no active bleeding is identified at repeat endoscopy, repeat endoscopy is performed due to (suspicion) of a delayed bleeding; therefore, this concerns a grade IIIa AE
**2025**	Balducci et al. [89]	3	Cholangitis	Repeat endoscopy for additional stent placement (stent through stent)	Unspecified, but ≤1 month	IIIa	IIIa	*NA*
		2	Hepatic abscess	Percutaneous drainage	Unspecified, but ≤1 month	IIIa	IIIa	*NA*
		2	Prosthesis dysfunction	Repeat endoscopy for additional stent placement (stent through stent)	Unspecified, but ≤1 month	IIIa	IIIa	*NA*
**2025**	Bronswijk et al. [91]	1	Post-procedural pain	Opioids for pain control	Unspecified	I	I	*NA*
		2	Post-ERCP pancreatitis	Medical treatment (not further specified) and 5 days of observation	Unspecified	II	II	*NA*
**2025**	Debourdeau et al. [93]	1	Biliary peritonitis due to LAMS dislodgement within an hour after the procedure	Surgical intervention and ICU admission	0 days	IVa	IVa	*NA*
		1	LAMS misplacement into the pancreatic duct in an elderly patient; family's refusal for further intervention	Death of the patient, attributed to progression of the disease	Unspecified	V	V	*NA*
		1	LAMS entering the peritoneum	Unmanageable endoscopically, percutaneous drainage and external drain placement did not ameliorate the septic condition from biliary peritonitis, death of the patient	Unspecified	V	V	*NA*
**2025**	Fortunati et al. [94]	3	Post-ERCP pancreatitis	Analgesics	Unspecified	II	I or II	If only analgesics are provided, this concerns a grade I AE – if the patient was also hospitalized for >24 hours, this concerns a grade II AE
		2	Escherichia coli sepsis	Antibiotics	Unspecified	II	II	*NA*
		1	Respiratory failure	Diuretics	Unspecified	II	II	*NA*

Endosc Int Open | DOI 10.1055/a-2965-6729 | © The Author(s).

		2	Sphincterotomy-related bleeding	Repeat endoscopy to establish hemostasis	Unspecified	IIIa	IIIa	*NA*
		1	Duodenal perforation	Repeat endoscopy for closure of the defect	Unspecified	IIIa	IIIa	*NA*
		1	Duodenal perforation	Surgery for closure of the defect	Unspecified	IIIb	IIIb	*NA*
		1	Gallbladder rupture	Surgery for cholecystectomy	Unspecified	IIIb	IIIb	*NA*
		1	Pulmonary oedema	ICU admission	Unspecified	IV	IVa or IVb	Based on the case description it is uncertain whether the patient suffered from single- or multiple organ failure, therefore this AE could either concern a grade IVa or IVb AE
		1	Cardiac arrest	Cardiopulmonary resuscitation performed, but death of the patient within 30 days after the procedure	Unspecified	V	V	*NA*
**2025**	Garg et al. [95]	1	Maldeployment of the LAMS	LAMS could not be endoscopically retrieved, surgical treatment (cholecystectomy)	Unspecified	IIIb	IIIb	*NA*
		1	Stent maldeployment leading to duodenal perforation	Duodenal stent placement and ERCP	Unspecified	IIIb	IIIa	Considering that only endoscopic intervention was required (no surgical intervention), this concerns a IIIa AE
		1	Bleeding	Radiological intervention (embolization)	Unspecified	IIIb	IIIa	Considering that only radiological intervention was required (no surgical intervention), this concerns a IIIa AE
**2025**	Gonzalez et al. [97]	1	Post-procedural bleeding	Repeat endoscopy without signs of active bleeding, blood transfusion, prolonged hospital stay >24 hours	Unspecified (discharge on post-operative day 6)	IIIa	IIIa	*NA*
**2025**	Ichkhanian et al. [98]	1	Bleeding	ICU admission, due to comorbidities no therapeutic measures were taken, death of the patient	0 days	V	V	*NA*
		2	Stent migration noted at routine repeat endoscopy	Removal of the stent, no further treatment given resolution of the abscess	Unspecified, but >2 days	I	No AE	See evaluation and recommendation as provided in Table 1 (Scenario 8)
		1	Gastrocolic fistula	Endoscopic suturing	Unspecified, but >2 days	IIIa	IIIa	*NA*

Endosc Int Open | DOI 10.1055/a-2965-6729 | © The Author(s).

**2025**	Lisotti et al. [101]	1	Bleeding	Prolonged hospital stay (undefined duration), but not requiring treatment	Unspecified	II	I or II	The case description provides no insights into the duration of the hospital admission: based on duration of the hospital stay (≤24 hours or >24 hours) this concerns either a grade I or II AE
		1	LAMS migration	(Urgent) endoscopic retrieval	Unspecified	IIIa	IIIa	*NA*
**2025**	Martinez-Moreno et al. [102]	1	Post-procedural bleeding caused by a mucosal laceration at the esophagogastric junction	Repeat endoscopy and closure with hemoclips	Unspecified	IIIa	IIIa	*NA*
**2025**	Neri et al. [104]	1	Sepsis	Antibiotics	Unspecified	II	II	*NA*
		2	Sepsis	Death of the patient	Unspecified	V	V	*NA*
**2025**	Pollack et al. [106]	1	Aspiration pneumonia	Hospital prolongation by 1 day	Unspecified	II	II	*NA*
		1	Refractory symptoms and difficulty of stent removal in a Billroth-I-related stricture	Surgical gastrojejunostomy	Unspecified	III	IIIb	*NA*
**2025**	Sarkis et al. [109]	1	(Suspected) perforation	Surgery	Unspecified	III	IIIb	Considering that surgical intervention was required, this AE concerns a grade IIIb AE
		2	Pneumomediastinum	ICU admission and prolongation of the hospital stay	Unspecified	IV	IVa or IVb	Based on the case description it is uncertain whether the patient suffered from single- or multiple organ failure, therefore this AE could either concern a grade IVa or IVb AE
		1	Inability to close the hypopharyngeal mucosal incision with clips	Requirement to place a covered stent	0 days (intraprocedural)	III	No AE	Presuming recovery without sequalae and no alterations to expected post-procedural course: see evaluation and recommendation as provided in Table 1 (Scenario 2)
		1	Perforation with a septal incision	Stent placement and a prolonged hospital admission (undefined duration)	0 days (intraprocedural)	III	I or II	The case description provides no insights into the duration of the hospital admission: based on duration of the hospital stay (≤24 hours or >24 hours) this concerns either a grade I or II AE
**2025**	Tachi et al. [111]	1	Fever and pain	Antipyretics	Unspecified	I	I	*NA*
		1	Cholecystitis	Treatment with antibiotics	Unspecified	II	II	*NA*

Endosc Int Open | DOI 10.1055/a-2965-6729 | © The Author(s).

**2025**	Terrin et al. [112]	1	Stent maldeployment	No need for defect closure; immediate repeated attempt successful	0 days (intraprocedural)	I	No AE	A Presuming recovery without sequalae and no alterations to expected post-procedural course: see evaluation and recommendation as provided in Table 1 (Scenario 2)
		1	Chronic fistula in patient with Douglas collections after colorectal surgery	Endoscopic closure	Unspecified	IIIa	IIIa	*NA*
		1	Chronic fistula in patient with Douglas collections after colorectal surgery	Surgical closure	Unspecified	IIIb	IIIb	*NA*
		2	Stent occlusion	Early endoscopic debridement	Unspecified	IIIa	IIIa	*NA*
		1	Small intra-abdominal abscess	Conservative treatment with antibiotics	Unspecified	II	II	*NA*
**2025**	Wannhoff et al. [113]	1	Gastrocolic fistula diagnosed when LAMS was pulled out (LAMS likely already traversed the colon during placement, but did not cause any symptoms)	Endoscopic intervention (placement of a fully-covered esophageal stent)	3 months	IIIa	IIIa	*NA*

AE, adverse event; NA, not applicable; ICU, intensive care unit; CT, computed tomography; EUS, endoscopic ultrasonography; LAMS, lumen-apposing metal stent; ERCP, endoscopic retrograde cholangiopancreatography; POEM, peroral endoscopic myotomy; CRP, C-reactive protein

Endosc Int Open | DOI 10.1055/a-2965-6729 | © The Author(s).

Table S4 Overview of the type of (likely) erroneous adverse event classifications using the AGREE classification

AGREE grade original article	AGREE grade consensus meeting	Frequency, n (%)
No AE	I	1 (3.8%)
I	No AE	14 (54%)
I	IIIa	1 (3.8%)
III	No AE	6 (23%)
III(a)	I/II	3 (12%)
IV	V	1 (3.8%)

Endosc Int Open | DOI 10.1055/a-2965-6729 | © The Author(s).

Table S5. Adverse event definition and period during which adverse events should be monitored according to different classifications

Classification	Definition of adverse event	Period during which adverse events should be monitored
**AGREE [114]**	"A negative outcome for the patient that prevents completion of a planned procedure or causes any deviation from the standard post-procedural course. Events during the procedure that did not change the standard post-procedural course are not regarded as adverse events. Adverse events should be recorded in an intention-to-scope manner, thus for all scheduled procedures."	"All adverse events occurring within 30 days after the procedure should be recorded as an adverse event, irrespective of the likelihood of a potential link between both events."
**ASGE [115]**	"An event that prevents completion of the planned procedure and/or results in admission to hospital, prolongation of existing hospital stay, another procedure (needing sedation/anesthesia), or subsequent medical consultation."	"Events can occur pre-procedure, intra-procedure (from entering the preparation area through leaving the endoscopy room), post-procedure (up to 14 days), and late (any time after 14 days). For delayed events, the number of days after the procedure should be documented."
**Clavien-Dindo [116,117]**	"Complications were defined as any deviation from the normal postoperative course. This definition also takes into account asymptomatic complications such as arrhythmia and atelectases."	"Any negative event occurring in a patient during hospital stay or within 30 days after surgery (whichever is longer), should be regarded as a surgical complication and should, therefore, be recorded"

Endosc Int Open | DOI 10.1055/a-2965-6729 | © The Author(s).

Figure S1 Sankey plot illustrates the characteristics of original research articles using the AGREE classification for grading (potential) adverse events related to gastrointestinal endoscopy.

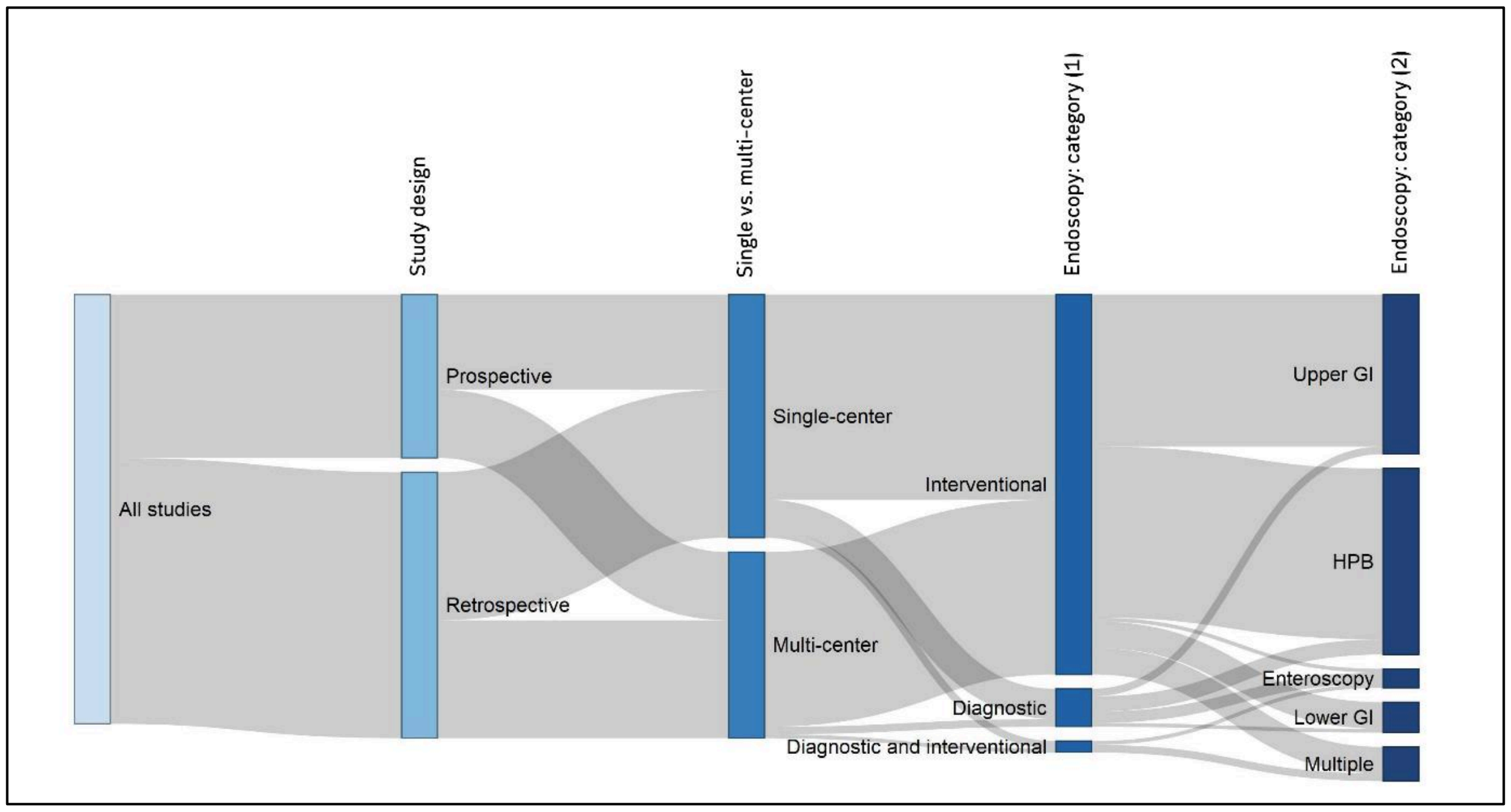

Endosc Int Open | DOI 10.1055/a-2965-6729 | © The Author(s).

APPENDIX

Appendix S1 Detailed description and evaluation of complex (adverse) event scenarios

Scenarios 1 to 8 were based on real-world scenarios as described within the literature. To improve clarity of scenarios, fictional details regarding involved patients (e.g., age, gender) and endoscopy procedures (e.g., indication) were added in case of missing information. Moreover, some (minor) alterations to the original AE scenarios were made in case this improved the scenarios' clarity or function as an illustrative example (e.g., "hospital stay prolongation by one day" was changed to "hospital stay prolongation <24 hours"). Scenario 9 involves a fictional scenario.

Scenario 1: intraprocedural event not requiring intervention, without sequelae
Based on a scenario as described by Colin et al. [5]

- **Scenario:** A 61-year-old male underwent antegrade motorized spiral enteroscopy for analysis of occult gastrointestinal blood loss. The procedure was performed under deep propofol sedation. During endoscope withdrawal, several superficial lacerations within the jejunum were observed. These were most likely caused by the rotations of the 'spirals' of the enteroscope. There were no signs of active bleeding or perforation and no intervention was required. Prolonged hospital stay for observation was not deemed required and the patient recovered without sequelae.

- **Evaluation:** As there were no sequelae for the patient or deviations from the standard post-procedural course, this event did not meet the AGREE classification criteria for an AE. Nevertheless, documentation of such unforeseen, non-consequential events may still be essential when assessing the safety profile of novel or emerging endoscopic techniques. Moreover, documenting such events may prove useful in evaluation of endoscopists' proficiency in specific procedures and techniques, or eligibility of selected patient groups to undergo certain procedures. Accordingly, following the precedent set by Crinò et al. [40], and in line with the ASGE recommendations [115], we recommend to document these events as "incidents".

Scenario 2: intraprocedural event requiring (additional) intervention, without sequelae
Based on a scenario as described by Geyl et al. [9]

- **Scenario:** A 72-year-old female with malignant distal biliary obstruction underwent endoscopic ultrasonography-guided choledochoduodenostomy (EUS-CDS). The procedure was performed under deep propofol sedation. Choledochoduodenostomy was performed using a electrocautery-enhanced lumen-apposing metal stent (ECE-LAMS). During the initial attempt to create the choledochoduodenal anastomosis, the distal flange of the stent was inadvertently deployed into the peritoneal cavity. The misdeployed stent was successfully retrieved. A second attempt during the same session, and using a new ECE-LAMS, was successful. The patient recovered without sequelae and there were no deviations from the standard post-procedural course (post-procedural observation was performed according to standard protocols).

Endosc Int Open | DOI 10.1055/a-2965-6729 | © The Author(s).

- **Evaluation:** Although this event necessitated an additional intraprocedural intervention, it did not result in sequelae for the patient or deviation from the standard post-procedural course. Therefore, this event does not meet the AGREE classification criteria for an AE. Nevertheless, consistent with the rationale provided with Scenario 1, documentation of such events may be relevant for evaluation of factors such as endoscopist proficiency. Accordingly, we recommend to report these events as "incidents". Had a repeat attempt been unfeasible or unsuccessful, thereby necessitating an additional interventional procedure (e.g., percutaneous drainage) or repeat endoscopy, this event should have been qualified differently (grade IIIa AE in case of percutaneous drainage or repeat endoscopy).

Scenario 3: intraprocedural event requiring (additional) endoscopic intervention, with sequelae
Based on a scenario as described by Falt et al. [8]

- **Scenario:** A 53-year-old male underwent retrograde motorized spiral enteroscopy for evaluation of suspected small bowel pathology identified on prior magnetic resonance imaging (MRI). The procedure was conducted under deep sedation with propofol. Endoscope insertion into the small intestine was uneventful. However, during withdrawal of the enteroscope, sigmoid intussusception occurred. Resolution was achieved through the parallel insertion of a pediatric colonoscope. The patient was hospitalized for observation (<24 hours), during which a single episode of self-limiting hematochezia was observed. No transfusion or pharmacologic intervention was required. After discharge, there were no further sequelae.

- **Evaluation:** As additional observation (<24 hours) was required, this event qualifies as a grade I AE. Had the intussusception presented post-procedurally and required a separate endoscopic intervention, this event should have been qualified as a grade IIIa AE.

Scenario 4: event occurring during a follow-up procedure (within 30 days following the initial procedure)
Based on a scenario as described by Stefanovic et al. [26]

- **Scenario:** A 48-year-old female with a medical history of a gastric bypass was diagnosed with a common bile duct stone. Endoscopic retrograde cholangiopancreatography (ERCP) for stone removal was indicated. To establish endoscopic access to the papilla, thereby allowing performance of the ERCP, an endoscopic ultrasound-guided gastrogastrostomy (EUS-GG) was performed using a lumen-apposing metal stent (LAMS). The ERCP was performed eight days following the EUS-GG. Both the EUS-GG and ERCP were performed under deep propofol sedation. During the ERCP procedure, the LAMS dislocated. This resulted in a gastric perforation. The ERCP procedure was discontinued, the LAMS was retrieved and the defect was closed from the stomach pouch using an over-the-scope clip. The patient was admitted for observation, during which the patient developed signs of peritonitis, most likely due to a persistent perforation. Laparoscopic surgical intervention was required to close the defect. Patient smoothly recovered from the surgical procedure, and was discharged two days after the procedure (with the common bile duct stone still in situ).

Endosc Int Open | DOI 10.1055/a-2965-6729 | © The Author(s).

- **Evaluation:** Although the event was contingent on the prior EUS-GG, the initial procedure was uneventful, and the AE occurred during the subsequent ERCP. As surgery was required, this event qualifies as a grade IIIb AE associated with the second procedure (the ERCP).

Scenario 5: event related to disfunction of endoscopically inserted materials

Based on a scenario as described by Chon et al. [38]

- **Scenario:** A 79-year-old male underwent endoscopic ultrasound-guided gallbladder drainage (EUS-GGD) for acute cholecystitis using a ECE-LAMS. The procedure was performed under deep sedation with propofol and was uneventful. Patient presented with signs of recurrent acute cholecystitis eleven days following the initial EUS-GD. Repeat endoscopy was performed, during which stent obstruction due to food impaction was observed. Balloon sweeping of the obstructed LAMS was performed, and an additional double-pigtail plastic stent was inserted. Patient smoothly recovered without further sequelae.

- **Evaluation:** Although (food-related) stent occlusion may be considered unavoidable, early (<30 days) stent dysfunction can also reflect suboptimal stent placement or positioning. Therefore, material-related events occurring within 30 days should be considered as AEs, irrespective of the presumed causality. As repeat endoscopy was required to dissolve stent occlusion in this scenario, this AE qualifies as a grade IIIa AE. In case the timeframe during which AEs were monitored and reported exceeds 30 days, we recommend to document (relevant) material-related events occurring after 30 days as “incidents”, rather than AEs. This recommendation is based on the decreasing likelihood that AEs occurring after 30 days are directly attributable to the (quality of the) endoscopic procedure initiated for material insertion. Instead, such events are more likely to reflect factors related to material performance, such as patency, and thus primarily offer insight into the long-term durability of the treatment or device, rather than the risk associated with the endoscopic procedure.

Scenario 6: intraprocedural event resulting in unforeseen intervention crossover

Based on a scenario as described by Koduri et al. [53]

- **Scenario:** A 56-year-old female with symptomatic walled-off necrosis (WON) after acute alcohol-induced pancreatitis underwent EUS-guided drainage of the WON. Drainage was performed in a study setting, in which the efficacy of EUS-guided drainage using either biflanged metal stents (BFMS) or plastic stents was compared. The patient was randomized to the plastic stent group. The procedure was performed under deep sedation with propofol. During the procedure, significant bleeding was observed at the puncture site. The bleeding was managed endoscopically. Considering that the BFMS was considered to offer better hemostatic potential, the endoscopist decided to crossover to perform WON drainage using a BFMS instead of a plastic stent. Post procedure, the patient was transfused with a single unit of whole blood and was discharged within 24 hours following the procedure. Patient recovered without further sequelae.

- **Evaluation:** Although the provided endoscopic intervention was unintended, no additional endoscopic procedures were required. However, there were sequelae for the patient. Considering these sequelae (need for blood transfusion), this event qualifies as a grade II AE.

Endosc Int Open | DOI 10.1055/a-2965-6729 | © The Author(s).

While the bleeding also drove the endoscopist's decision for unscheduled intervention crossover, the intervention crossover was not associated with any sequelae for the patient. Accordingly, in case no blood transfusion and/or prolonged hospital stay had been required, this event would not have met the AGREE criteria for an AE. Notwithstanding, consistent with the rationale as provided with Scenario 1, we recommend documentation of such events (unscheduled intervention crossover) as "incidents".

<u>Scenario 7: detection of abnormalities during a (routine) follow-up procedure</u>

Based on a scenario as described by Nimri et al. [65]

- **Scenario:** A 65-year-old male with a medical history of liver cirrhosis underwent EUS-guided drainage of a pancreatic fluid collection (cyst) using a hot LAMS, along with two plastic stents through the LAMS. During routine (scheduled) follow-up esophagogastroduodenoscopy, the stent was found to have migrated distally into the cyst. The patient was asymptomatic. The LAMS was retrieved and a new LAMS was deployed. Eventually, the fluid collection completely resolved and the LAMS was removed (45 days following the initial procedure).

- **Evaluation:** As the stent migration was detected during a routine (scheduled) follow-up procedure and there were no sequelae for the patient, this event does not meet the AGREE criteria for an AE. However, consistent with the rationale as provided with Scenario 1, the stent migration may be documented as an "incident". Had the patient developed symptoms requiring unscheduled endoscopy, this event should have been classified as a grade IIIa AE.

<u>Scenario 8: a procedure associated with an event with an onset within 30 days and evolving clinical consequences beyond 30 days</u>

Based on a scenario as described by Rodriguez de Santiago et al. [68]

- **Scenario:** A 78-year-old male underwent endoscopic balloon dilatation of the esophagus due to a stenosis related to a previous endoscopic mucosal resection. The dilation was performed without immediate signs of adverse events. However, within an hour post-procedure, the patient developed increasing retrosternal chest pain and nausea. Computed tomography imaging of the thorax showed signs consistent with an esophageal perforation. Repeat endoscopy was performed for placement of a fully covered stent, and patient was admitted and treated with antibiotics, intravenous fluids and dietary restrictions. Both procedures were performed under deep sedation with propofol. Post procedure, patient remained hemodynamically stable and showed steady clinical improvement over the next few days. The patient was discharged after six days. After discharge, the patient was closely monitored and elective stent removal was scheduled after six weeks.

  Five weeks following the dilatation, the patient presented to the emergency department with worsening dyspnea and low-grade fever. Chest CT revealed a bronchoesophageal fistula. Additionally, patient showed signs of heart failure. The patient's condition deteriorated quickly. Given the patient's general condition and the complexity of the fistula, surgical intervention was deemed too high-risk. Conservative management was pursued. Despite maximal medical therapy, the patient's condition continued to deteriorate and the patient passed away 53 days after the dilatation.

Endosc Int Open | DOI 10.1055/a-2965-6729 | © The Author(s).

- **Evaluation:** According to the AGREE classification recommendations, all (potential) AEs with an onset within 30 days post-procedure must be documented. In this scenario, an esophageal perforation was identified and treated with a repeat endoscopy for stent placement within this 30-days period. Although the fistula, ultimately leading to the patient's death, was diagnosed beyond the 30-days timeframe, it was directly related to the perforation (and subsequent stent placement) diagnosed shortly following the dilatation. Following the AGREE classification recommendations, when multiple adverse events occur following the same endoscopic procedure and are clearly related (e.g., perforation and fistula), the event with the most severe consequences should be considered. As the fistula eventually resulted in the patient's death, this event qualifies as a Grade V AE.

Scenario 9: event potentially related to a scheduled endoscopic procedure

Fictional scenario

- **Scenario:** A 67-year-old female attended the hospital for surveillance colonoscopy after previous removal of a non-malignant 25 mm polyp in the ascending colon. The patient had taken bowel preparation according to prescription, starting the day before the procedure. While in the endoscopy unit holding area, during the placement of an intravenous catheter, patient experienced and unexpected cardiac arrest. Multiple defibrillation attempts and advanced resuscitative measures were unsuccessful.

- **Evaluation:** According to the AGREE recommendations, adverse events should be recorded in an intention-to-scope manner. This includes events occurring during preparation for endoscopic procedures. Although the causality between the preparation for colonoscopy (e.g., bowel preparation) and the cardiac arrest is uncertain, this event should be recorded as an AE potentially related to the (preparation for the) colonoscopy. Similar rationale applies to events occurring during, for example, preprocedural sedation. As the cardiac arrest resulted in the patient's death, the AE qualifies as a grade V AE.

Endosc Int Open | DOI 10.1055/a-2965-6729 | © The Author(s).

Appendix S2 Reference list for Figures 4A-B

Figure 4A:

- de Rauglaudre B, Pioche M, Caillol F et al. Follow-up after endoscopic resection for early gastric cancer in 3 French referral centers. iGIE 2022; 1: 49-56. doi:10.1016/j.igie.2022.10.004
- Crinò SF, Napoleon B, Facciorusso A et al. Endoscopic Ultrasound-guided Radiofrequency Ablation Versus Surgical Resection for Treatment of Pancreatic Insulinoma. Clin Gastroenterol Hepatol 2023; 21: 2834-2843.e2832. doi:10.1016/j.cgh.2023.02.022
- Hanssens M, Dhondt E, Degroote H et al. EUS-guided versus PTC-guided rendezvous in case of failed ERCP: a case-control study. Surg Endosc 2023; 37: 3492-3497. doi:10.1007/s00464-022-09827-1
- Kucha P, Zorniak M, Szmit M et al. To push or to pull? A clinical audit on the efficacy and safety of the pull and push percutaneous endoscopic gastrostomy techniques in oncological patients. United European Gastroenterology J 2023; 11: 951-959. doi:10.1002/ueg2.12467
- Monino L, Pérez-Cuadrado-Robles E, Gonzalez J-M et al. Endoscopic ultrasound-guided gastroenterostomy with lumen-apposing metal stents: A retrospective multicentric comparison of wireless and over-the-wire techniques. Endoscopy 2023; 55: 991-999. doi:10.1055/a-2119-7529
- Bekaert J, Van Langendonck S, Van Heddegem N et al. Performance of novices in Endoscopic Submucosal Dissection starting directly in human patients under direct supervision of an expert endoscopist. Acta Gastroenterol Belg 2024; 87: 478-483. doi:10.51821/87.4.13604
- Crinò SF, Zorzi A, Tavian P et al. Same versus separate sessions of endoscopic ultrasound-guided fine-needle biopsy and endoscopic retrograde cholangiopancreatography for distal malignant biliary obstruction: a propensity score-matched study. Expert Rev Gastroenterol Hepatol 2024; 18: 551-559. doi:10.1080/17474124.2024.2399176
- Giordano A, Compañy L, Alajarin-Cervera M et al. Prospective multicenter study to identify optimal target population for motorized spiral enteroscopy. Sci Rep 2024; 14: 16788. doi:10.1038/s41598-024-64510-w
- Gombošová L, Deptová J, Jochmanová I et al. Endoscopic Complications Are More Frequent in Levodopa–Carbidopa Intestinal Gel Treatment via JET-PEG in Parkinson's Disease Patients Compared to Nutritional PEG in Non-Parkinson's Disease Patients. J Clin Med 2024; 13: 703. doi:10.3390/jcm13030703
- Goubert K, Degroote H, De Vos M et al. Factors Associated with the Severity of ERCP-Related Complications: A Retrospective Single-Centre Study. J Clin Med 2024; 13: 7481. doi:10.3390/jcm13237481
- Monino L, Marique L, Deswysen Y et al. Endoscopic Management of Biliary and Pancreatic Pathologies in Roux-en-Y Gastric Bypass Patients: Development of a Treatment Algorithm Based on 9-Year Experience. Obes Surg 2024; 34: 3717-3725. doi:10.1007/s11695-024-07471-3
- Steinbrück I, Ebigbo A, Kuellmer A et al. Cold Versus Hot Snare Endoscopic Resection of Large Nonpedunculated Colorectal Polyps: Randomized Controlled German CHRONICLE Trial. Gastroenterology 2024; 167: 764-777. doi:10.1053/j.gastro.2024.05.013
- Steinbrück I, Rempel V, Kuellmer A et al. Flexible endoscopic treatment of Zenker's diverticulum—a retrospective, observational multicenter study. Surg Endosc 2024; 38: 6493-6505. doi:10.1007/s00464-024-11234-7
- Vauquelin B, Quénéhervé L, Pioche M et al. Factors associated with early failure of peroral endoscopic myotomy in achalasia. Gastrointest Endosc 2024; 99: 349-357.e343. doi:10.1016/j.gie.2023.10.025
- Debourdeau A, Daniel J, Caillo L et al. Effectiveness of endoscopic ultrasound (EUS)-guided choledochoduodenostomy vs. EUS-guided gallbladder drainage for jaundice in patients with malignant distal biliary obstruction after failed endoscopic retrograde

cholangiopancreatography: Retrospective, multicenter study (GALLBLADEUS Study). Dig Endosc 2025; 37: 103-114. doi:10.1111/den.14750

- Gökce E, Devisscher L, Rashidian N et al. Lumen-apposing metal stents for anastomosis creation throughout the gastrointestinal tract: A large single-center experience. DEN open 2025; 5: e419. doi:10.1002/deo2.419
- Kadkhodayan K, Pathak S, Chandan S et al. EUS-guided rendezvous is a viable salvage technique for failed billiary cannulation in patients with Roux-en-Y gastric bypass undergoing BAE-ERCP. Endosc Int Open 2025; 13: a25097500. doi:10.1055/a-2509-7500
- Romanczyk M, Klimkowski R, Kukla M et al. Safety, efficacy and changing trends in endoscopic ablation for dysplastic Barrett's esophagus in Poland: a long-term multicenter retrospective analysis. Pol Arch Intern Med 2025; 135: 16912. doi:10.20452/pamw.16912

Figure 4B:

- Pérez-Cuadrado-Robles E, Alric H, Aidibi A et al. EUS-Guided Gastroenterostomy in Malignant Gastric Outlet Obstruction: A Comparative Study between First- and Second-Line Approaches after Enteral Stent Placement. Cancers (Basel) 2022; 14: 5516. doi:10.3390/cancers14225516
- Colin H, Donati A, Monino L et al. Motorized spiral enteroscopy: effectiveness when used for new indications. Acta Gastroenterol Belg 2023; 86: 269-275. doi:10.51821/86.2.11779
- Rughwani H, Singh AP, Ramchandani M et al. A Randomized, Controlled Trial Comparing the Total Enteroscopy Rate and Diagnostic Efficacy of Novel Motorized Spiral Enteroscopy and Single-Balloon Enteroscopy in Patients With Small-Bowel Disorders: The Motor Trial (NCT 05548140). Am J Gastroenterol 2023; 118: 1855-1863. doi:10.14309/ajg.0000000000002409
- Tejedor-Tejada J, Hermida B, Pipa-Muniz M et al. Feasibility and Safety of Through-the-scope versus Over-the-wire Stents for the Palliation of Malignant Dysphagia. A Cohort Real- Life Study. J Gastrointestin Liver Dis 2023; 32: 143-149. doi:10.15403/jgld-4815
- Yzet C, Le Baleur Y, Albouys J et al. Use of endoscopic submucosal dissection or full-thickness resection device to treat residual colorectal neoplasia after endoscopic resection: a multicenter historical cohort study. Endoscopy 2023; 55: 1002-1009. doi:10.1055/a-2116-9930
- Atay A, Yuksel I. The Experience of a Tertiary Referral Center with Endoscopic Management and Combining Percutaneous Intervention for the Treatment of Walled-Off Necrosis: A Stepwise Approach. J Clin Med 2024; 13: 4916. doi:10.3390/jcm13164916
- Barras J, Poncin M, Van Severen C et al. G-POEM in Belgium: a retrospective study. Acta Gastroenterol Belg 2024; 87: 469-477. doi:10.51821/87.4.13463
- Conti Bellocchi MC, Gasparini E, Stigliano S et al. Endoscopic Ultrasound-Guided Gastroenterostomy versus Enteral Stenting for Malignant Gastric Outlet Obstruction: A Retrospective Propensity Score-Matched Study. Cancers 2024; 16: 724. doi:10.3390/cancers16040724
- Gibiino G, Binda C, Papparella LG et al. Technical failure during colorectal endoscopic full-thickness resection: the "through thick and thin" study. Endoscopy 2024; 56: 831-839. doi:10.1055/a-2328-4753
- Gloor S, Minder S, Schnell B et al. Antegrade balloon dilatation of the duodenal papilla during laparoscopic cholecystectomy versus endoscopic retrograde cholangiography in patients with acute choledocholithiasis: a case control matched study. Surg Endosc 2024; 38: 3810-3818. doi:10.1007/s00464-024-10909-5
- Katrevula A, Singla N, Rughwani H et al. Comprehensive audit of gastrointestinal endoscopy adverse events: Emphasizing quality indicators and economic impact with the AIG-AGREE modification. Endosc Int Open 2024; 12: E1242-E1250. doi:10.1055/a-2435-5445
- Ligresti D, Binda C, Fugazza A et al. Direct endoscopic necrosectomy: Pilot study of a new dedicated device. Endosc Int Open 2024; 12: E962-E967. doi:10.1055/a-2376-1913

- Mangiavillano B, Moon JH, Facciorusso A et al. Endoscopic ultrasound-guided gallbladder drainage as a first approach for jaundice palliation in unresectable malignant distal biliary obstruction: Prospective study. Dig Endosc 2024; 36: 351-358. doi:10.1111/den.14606
- Mangiavillano B, Ramai D, Kahaleh M et al. Outcomes Of Lumen Apposing Metal Stent Placement In Patients With Surgically Altered Anatomy: A Multicenter International Experience. Endosc Int Open 2024; 12: E1143-1149. doi:10.1055/a-2411-1814
- Pérez-Cuadrado-Robles E, Phillpotts S, Bronswijk M et al. PERcutaneous transhepatic CHOLangioscopy using a new single-operator short cholangioscope (PERCHOL): European feasibility study. Dig Endosc 2024; 36: 719-725. doi:10.1111/den.14697
- Tejedor-Tejada J, Alvarez-Alvarez A, Olmos JM et al. Secondary uncovered versus fully-covered metal stents for the management of occluded stent in unresectable distal malignant biliary obstruction. Gastroenterol Hepatol 2024; 47: 502218. doi:10.1016/j.gastrohep.2024.502218
- Van Severen C, Koch S, Faure J et al. Peroral endoscopic myotomy: a two-center retrospective study of practice and adverse events. Acta Gastroenterol Belg 2024; 87: 7-13. doi:10.51821/87.1.12358
- Verhoeve S, Verloop C, Bruno M et al. Efficacy of EUS-guided keyhole biopsies in diagnosing subepithelial lesions of the upper gastrointestinal tract. Endosc Int Open 2024; 12: E1183-e1189. doi:10.1055/a-2417-0580
- Wilson N, Abdallah M, Schulman AR et al. Outcomes of cold snare EMR of nonampullary duodenal adenomas ≥ 1 cm: a multicenter study. Gastrointest Endosc 2024; 99: 971-980.e971. doi:10.1016/j.gie.2023.12.007
- Beunon C, Debourdeau A, Schaefer M et al. Technical failure of endoscopic ultrasound choledocoduodenostomy: multicenter case-control study on rescue techniques, consequences and risk factors. Endoscopy 2025; Online ahead of print. doi:10.1055/a-2541-2973
- Fortunati F, Monino L, Deprez P et al. Endoscopic retrograde cholangiopancreatography in patients with different types of total and partial gastrectomy. Acta Gastroenterol Belg 2025; 88: 23-30. doi:10.51821/88.1.13779
- Garg R, Baron T, Trieu JA et al. Safety and Effectiveness of EUS-Guided Gallbladder Drainage in Patients with Cirrhosis: An International Multicenter Experience. Endoscopy 2025; 57: 593-601. doi:10.1055/a-2517-0927
- Gonzalez J-M, Phelip J, Gasmi M et al. Endoscopic Ultrasound Guided Gastroenterostomy for managing gastroparesis refractory to gastric peroral endoscopic pylorotomy: a new promising therapeutic option. iGIE 2025; 4: 16-20. doi:10.1016/j.igie.2025.01.011
- Ichkhanian Y, Chaudhary AJ, Veracruz N et al. Endoscopic Ultrasound-Guided Drainage of Intra-Abdominal Abscess Using 15-mm vs. 10-mm Lumen-Apposing Metal Stents: An International Case-Matched Study. Gastrointest Endosc 2025; Online ahead of print. doi:10.1016/j.gie.2025.01.002
- Lisotti A, D'Errico F, Fusaroli P et al. Safety and efficacy of endoscopic ultrasound-guided pelvic abscess drainage with lumen-apposing metal stents for complicated acute diverticulitis. Gastrointest Endosc 2025; Online ahead of print. doi:10.1016/j.gie.2025.02.003
- Sarkis Y, Stainko S, Perkins A et al. Comparison of flexible endoscopic needle-knife septotomy and peroral endoscopic myotomy for treatment of Zenker's diverticulum. Gastrointest Endosc 2025; 101 82-89. doi:10.1016/j.gie.2024.08.035
- Terrin M, D’Errico F, Rotkopf H et al. First-intention EUS-guided transluminal drainage with LAMS: an effective and safe method for management of fluid collections after any kind of surgery. Surg Endosc 2025; 39: 2415-2424. doi:10.1007/s00464-025-11615-6
- Tachi K, Hara K, Okuno N et al. Efficacy and safety of endoscopic ultrasound-guided hepaticogastrostomy for biliary drainage in hypervascular hepatocellular carcinoma. Clin Endosc 2025; 58: 448-456. doi:10.5946/ce.2024.079

- Wannhoff A, Canakis A, Sharaiha RZ et al. Endoscopic ultrasound-guided gastroenterostomy for the treatment of gastric outlet obstruction secondary to acute pancreatitis. Endoscopy 2025; 57: 249-254

Endosc Int Open | DOI 10.1055/a-2965-6729 | © The Author(s).

SUPPLEMENTARY MATERIALS - REFERENCES

1. Pérez-Cuadrado-Robles E, Alric H, Aidibi A et al. EUS-Guided Gastroenterostomy in Malignant Gastric Outlet Obstruction: A Comparative Study between First- and Second-Line Approaches after Enteral Stent Placement. Cancers (Basel) 2022; 14: 5516. doi:10.3390/cancers14225516
2. de Rauglaudre B, Pioche M, Caillol F et al. Follow-up after endoscopic resection for early gastric cancer in 3 French referral centers. iGIE 2022; 1: 49-56. doi:10.1016/j.igie.2022.10.004
3. Aelvoet AS, Karstensen JG, Bastiaansen BAJ et al. Cold snare polypectomy for duodenal adenomas in familial adenomatous polyposis: a prospective international cohort study. Endosc Int Open 2023; 11: E1056-e1062. doi:10.1055/a-2165-7436
4. Borrelli de Andreis F, Boskoski I, Mascagni P et al. Safety and efficacy of endoscopic ultrasound-guided radiofrequency ablation for pancreatic insulinoma: A single-center experience. Pancreatology 2023; 23: 543-549. doi:10.1016/j.pan.2023.05.004
5. Colin H, Donati A, Monino L et al. Motorized spiral enteroscopy: effectiveness when used for new indications. Acta Gastroenterol Belg 2023; 86: 269-275. doi:10.51821/86.2.11779
6. Crinò SF, Napoleon B, Facciorusso A et al. Endoscopic Ultrasound-guided Radiofrequency Ablation Versus Surgical Resection for Treatment of Pancreatic Insulinoma. Clin Gastroenterol Hepatol 2023; 21: 2834-2843.e2832. doi:10.1016/j.cgh.2023.02.022
7. Crispino F, Merola E, Tasini E et al. Adverse events in gastrointestinal endoscopy: Validation of the AGREE classification in a real-life 5-year setting. Dig Liver Dis 2023; 55: 933-937. doi:10.1016/j.dld.2023.04.007
8. Falt P, Urban O. Motorized spiral enteroscopy - a prospective analysis of 82 procedures at a single tertiary center. Scand J Gastroenterol 2023; 58: 1207-1212. doi:10.1080/00365521.2023.2212311
9. Geyl S, Redelsperger B, Yzet C et al. Risk factors for stent dysfunction during long-term follow-up after EUS-guided biliary drainage using lumen-apposing metal stents: A prospective study. Endosc Ultrasound 2023; 12: 237-244. doi:10.4103/EUS-D-22-00120
10. Ghazi R, AbiMansour JP, Mahmoud T et al. Uncovered versus fully covered self-expandable metal stents for the management of distal malignant biliary obstruction. Gastrointest Endosc 2023; 98: 577-584. doi:10.1016/j.gie.2023.05.047
11. Hanssens M, Dhondt E, Degroote H et al. EUS-guided versus PTC-guided rendezvous in case of failed ERCP: a case-control study. Surg Endosc 2023; 37: 3492-3497. doi:10.1007/s00464-022-09827-1
12. Hayat M, Schlachterman A, Schiavone G et al. Initial multicenter experience using a novel articulating through-the-scope traction device for endoscopic submucosal dissection. Endoscopy International Open 2023; 11: E778-E784. doi:10.1055/a-2117-8444
13. Jaroenlapnopparat A, Thompson CC, Jirapinyo P. Effect of Transoral Outlet Reduction on Hepatic Fibrosis in Roux-en-Y Gastric Bypass Patients with Weight Regain and Non-alcoholic Fatty Liver Disease. Obes Surg 2023; 33: 2303-2310. doi:10.1007/s11695-023-06668-2
14. Khan HM, Brar TS, Hasan MK et al. Prospective study on the efficacy of endoscopic through-the-scope tack and suture system for gastric peroral endoscopic myotomy mucosal incision site closure. Endosc Int Open 2023; 11: E187-E192. doi:10.1055/a-1978-6842
15. Kucha P, Zorniak M, Szmit M et al. To push or to pull? A clinical audit on the efficacy and safety of the pull and push percutaneous endoscopic gastrostomy techniques in oncological patients. United European Gastroenterology J 2023; 11: 951-959. doi:10.1002/ueg2.12467
16. Liu W-H, Huang X-Y, Hu X et al. Initial experience of visualized biliary cannulation during ERCP. Endoscopy 2023; 55: 1037-1042. doi:10.1055/a-2113-8952
17. Mangiavillano B, Larghi A, Vargas-Madrigal J et al. EUS-guided gastroenterostomy using a novel electrocautery lumen apposing metal stent for treatment of gastric outlet obstruction (with video). Dig Liver Dis 2023; 55: 644-648. doi:10.1016/j.dld.2023.02.009

Endosc Int Open | DOI 10.1055/a-2965-6729 | © The Author(s).

18. Momblan D, Garcia AZG, Busquets D et al. Endoscopic vacuum therapy for upper gastrointestinal leaks and perforations: Analysis from a multicenter Spanish registry. Am J Gastroenterol 2023; 118: 1797-1806. doi:10.14309/ajg.0000000000002475
19. Monino L, Pérez-Cuadrado-Robles E, Gonzalez J-M et al. Endoscopic ultrasound-guided gastroenterostomy with lumen-apposing metal stents: A retrospective multicentric comparison of wireless and over-the-wire techniques. Endoscopy 2023; 55: 991-999. doi:10.1055/a-2119-7529
20. Mutignani M, Forti E, Pugliese F et al. Endoscopic entero-enteral bypass to treat postsurgical benign complications of hepatico-jejunostomy: Update of a 7-year single-center experience. Endoscopy International Open 2023; 11: E394-E400. doi:10.1055/a-2032-3077
21. Nakamura J, Ogura T, Ueno S et al. Liver impaction technique improves technical success rate of guidewire insertion during EUS-guided hepaticogastrostomy (with video). Therap Adv Gastroenterol 2023; 16: 17562848231188562. doi:10.1177/17562848231188562
22. Napoléon B, Lisotti A, Caillol F et al. Risk factors for EUS-guided radiofrequency ablation adverse events in patients with pancreatic neoplasms: a large national French study (RAFPAN study). Gastrointest Endosc 2023; 98: 392-399. e391. doi:10.1016/j.gie.2023.04.003
23. Pattynama LMD, Eshuis WJ, van Berge Henegouwen MI et al. Vacuum-stent: A combination of endoscopic vacuum therapy and an intraluminal stent for treatment of esophageal transmural defects. Front Surg 2023; 10: 1145984. doi:10.3389/fsurg.2023.1145984
24. Pérez-Cuadrado-Robles E, Alric H, Queneherve L et al. Risk factors of anastomosis-related difficult endoscopic retrograde cholangiopancreatography following endoscopic ultrasound-guided gastro-gastrostomy using a standardized protocol (with video). Dig Endosc 2023; 35: 909-917. doi:10.1111/den.14544
25. Rughwani H, Singh AP, Ramchandani M et al. A Randomized, Controlled Trial Comparing the Total Enteroscopy Rate and Diagnostic Efficacy of Novel Motorized Spiral Enteroscopy and Single-Balloon Enteroscopy in Patients With Small-Bowel Disorders: The Motor Trial (NCT 05548140). Am J Gastroenterol 2023; 118: 1855-1863. doi:10.14309/ajg.0000000000002409
26. Stefanovic S, Degroote H, Hindryckx P. Reduction of Lams-Related Adverse Events with Accumulating Experience in a Large-Volume Tertiary Referral Center. J Clin Med 2023; 12: 1037. doi:10.3390/jcm12031037
27. Tejedor-Tejada J, Hermida B, Pipa-Muniz M et al. Feasibility and Safety of Through-the-scope versus Over-the-wire Stents for the Palliation of Malignant Dysphagia. A Cohort Real- Life Study. J Gastrointestin Liver Dis 2023; 32: 143-149. doi:10.15403/jgld-4815
28. Tiebie E, Baerends E, Boeije T et al. Efficacy of cola ingestion for oesophageal food bolus impaction: open label, multicentre, randomised controlled trial. The BMJ 2023; 383: e077294. doi:10.1136/bmj-2023-077294
29. Weiss S, Pellat A, Corre F et al. Predictive factors of radiofrequency ablation failure in the treatment of dysplastic Barrett's esophagus. Clin Res Hepatol Gastroenterol 2023; 47: 102065. doi:10.1016/j.clinre.2022.102065
30. Wessels EM, Schuitenmaker JM, Bastiaansen BAJ et al. Efficacy and safety of peroral endoscopic myotomy for esophageal diverticula. Endosc Int Open 2023; 11: E546-E552. doi:10.1055/a-2071-6744
31. Yzet C, Le Baleur Y, Albouys J et al. Use of endoscopic submucosal dissection or full-thickness resection device to treat residual colorectal neoplasia after endoscopic resection: a multicenter historical cohort study. Endoscopy 2023; 55: 1002-1009. doi:10.1055/a-2116-9930
32. Abad J, Llop E, Arias-Loste MT et al. Endoscopic sleeve gastroplasty plus lifestyle intervention in patients with MASH: a multicentre, sham-controlled, randomized trial. Clin Gastroenterol Hepatol 2024; Online ahead of print. doi:10.1016/j.cgh.2024.10.027
33. Albers D, Witt M, Pandolfino JE et al. Peroral endoscopic myotomy for hypercontractile (Jackhammer) esophagus: A retrospective multicenter series with long-term follow-up. United European Gastroenterol J 2024; 12: 930-940. doi:10.1002/ueg2.12586

Endosc Int Open | DOI 10.1055/a-2965-6729 | © The Author(s).

34. Atay A, Yuksel I. The Experience of a Tertiary Referral Center with Endoscopic Management and Combining Percutaneous Intervention for the Treatment of Walled-Off Necrosis: A Stepwise Approach. J Clin Med 2024; 13: 4916. doi:10.3390/jcm13164916
35. Barras J, Poncin M, Van Severen C et al. G-POEM in Belgium: a retrospective study. Acta Gastroenterol Belg 2024; 87: 469-477. doi:10.51821/87.4.13463
36. Bekaert J, Van Langendonck S, Van Heddegem N et al. Performance of novices in Endoscopic Submucosal Dissection starting directly in human patients under direct supervision of an expert endoscopist. Acta Gastroenterol Belg 2024; 87: 478-483. doi:10.51821/87.4.13604
37. Castro JSL, Taglieri E, Neto OM et al. Clinical usefulness of tissue acquisition of pancreatic cystic lesions using an endoscopic ultrasound-guided needle for histological analysis. Endosc Int Open 2024; 12: E155-E163. doi:10.1055/a-2240-0678
38. Chon HK, Lee YC, Kim TH et al. Revolutionizing outcomes: endoscopic ultrasound-guided gallbladder drainage using innovative electrocautery enhanced-lumen apposing metal stents for high-risk surgical patients. Scientific Reports 2024; 14: 12893. doi:10.1038/s41598-024-63608-5
39. Conti Bellocchi MC, Gasparini E, Stigliano S et al. Endoscopic Ultrasound-Guided Gastroenterostomy versus Enteral Stenting for Malignant Gastric Outlet Obstruction: A Retrospective Propensity Score-Matched Study. Cancers 2024; 16: 724. doi:10.3390/cancers16040724
40. Crinò SF, Zorzi A, Tavian P et al. Same versus separate sessions of endoscopic ultrasound-guided fine-needle biopsy and endoscopic retrograde cholangiopancreatography for distal malignant biliary obstruction: a propensity score-matched study. Expert Rev Gastroenterol Hepatol 2024; 18: 551-559. doi:10.1080/17474124.2024.2399176
41. Dell’Anna G, Mandarino FV, Fanizza J et al. Endoscopic Management of Post-Esophagectomy Delayed-Gastric-Conduit-Emptying (DGCE): Results from a Cohort Study in a Tertiary Referral Center with Comparison between Procedures. Cancers (Basel) 2024; 16: 3457. doi:10.3390/cancers16203457
42. Drews J, Baar LC, Schmeisl T et al. Biliary drainage in palliative and curative intent European patients with hilar cholangiocarcinoma and malignant hilar obstruction: a retrospective single center analysis. BMC Gastroenterol 2024; 24: 359. doi:10.1186/s12876-024-03429-y
43. Fritzsche JA, Fockens P, Besselink MG et al. Endoscopic ultrasound-guided choledochoduodenostomy using single-step lumen-apposing metal stents for primary drainage of malignant distal biliary obstruction (SCORPION-p): a prospective pilot study. Endoscopy 2024; 56: 47-52. doi:10.1055/a-2134-3537
44. Fritzsche JA, Fockens P, Besselink MG et al. Optimizing EUS-guided choledochoduodenostomy with lumen-apposing metal stents for primary drainage of malignant distal biliary obstruction (SCORPION-IIp): a prospective pilot study. Gastrointest Endosc 2024; 101: 1009-1016. doi:10.1016/j.gie.2024.10.012
45. Gibiino G, Binda C, Papparella LG et al. Technical failure during colorectal endoscopic full-thickness resection: the “through thick and thin” study. Endoscopy 2024; 56: 831-839. doi:10.1055/a-2328-4753
46. Giordano A, Compañy L, Alajarin-Cervera M et al. Prospective multicenter study to identify optimal target population for motorized spiral enteroscopy. Sci Rep 2024; 14: 16788. doi:10.1038/s41598-024-64510-w
47. Gloor S, Minder S, Schnell B et al. Antegrade balloon dilatation of the duodenal papilla during laparoscopic cholecystectomy versus endoscopic retrograde cholangiography in patients with acute choledocholithiasis: a case control matched study. Surg Endosc 2024; 38: 3810-3818. doi:10.1007/s00464-024-10909-5
48. Gombošová L, Deptová J, Jochmanová I et al. Endoscopic Complications Are More Frequent in Levodopa–Carbidopa Intestinal Gel Treatment via JET-PEG in Parkinson’s Disease Patients Compared to Nutritional PEG in Non-Parkinson’s Disease Patients. J Clin Med 2024; 13: 703. doi:10.3390/jcm13030703

Endosc Int Open | DOI 10.1055/a-2965-6729 | © The Author(s).

49. Goubert K, Degroote H, De Vos M et al. Factors Associated with the Severity of ERCP-Related Complications: A Retrospective Single-Centre Study. J Clin Med 2024; 13: 7481. doi:10.3390/jcm13237481
50. Jürgensen C, Eckart M, Haberbosch L et al. Endoscopic ultrasound-guided ethanol ablation versus surgical resection of insulinomas. Ultraschall Med 2024; 45: 519-527. doi:10.1055/a-2204-5814
51. Katrevula A, Singla N, Rughwani H et al. Comprehensive audit of gastrointestinal endoscopy adverse events: Emphasizing quality indicators and economic impact with the AIG-AGREE modification. Endosc Int Open 2024; 12: E1242-E1250. doi:10.1055/a-2435-5445
52. Khalaf MA, Ayoub F, Staggers KA et al. Learning curve for endoscopic submucosal dissection (ESD) in the United States: a large, untutored single-operator experience. Endoscopy International Open 2024; 12: E905-E913. doi:10.1055/a-2337-3865
53. Koduri KK, Jagtap N, Lakhtakia S et al. Biflanged metal stents versus plastic stents for endoscopic ultrasound-guided drainage of walled-off necrosis: a randomized controlled trial. Endoscopy 2024; 56: 915-923. doi:10.1055/a-2332-3448
54. Ligresti D, Binda C, Fugazza A et al. Direct endoscopic necrosectomy: Pilot study of a new dedicated device. Endosc Int Open 2024; 12: E962-E967. doi:10.1055/a-2376-1913
55. Lisotti A, Cominardi A, Conti Bellocchi MC et al. Repeated endoscopic ultrasound-guided fine-needle biopsy of solid pancreatic lesions after previous nondiagnostic or inconclusive sampling. Dig Endosc 2024; 36: 615-624. doi:10.1111/den.14686
56. Mangiavillano B, Moon JH, Facciorusso A et al. Endoscopic ultrasound-guided gallbladder drainage as a first approach for jaundice palliation in unresectable malignant distal biliary obstruction: Prospective study. Dig Endosc 2024; 36: 351-358. doi:10.1111/den.14606
57. Mangiavillano B, Ramai D, Kahaleh M et al. Outcomes Of Lumen Apposing Metal Stent Placement In Patients With Surgically Altered Anatomy: A Multicenter International Experience. Endosc Int Open 2024; 12: E1143-1149. doi:10.1055/a-2411-1814
58. Masciangelo G, Cecinato P, Bacchilega I et al. Urgent ERCP performed with single-use duodenoscope (SUD) in patients with moderate-to-severe cholangitis: Single-center prospective study. Endosc Int Open 2024; 12: E116-E122. doi:10.1055/a-2219-0826
59. Mesureur L, Deprez PH, Bisschops R et al. Safety and efficacy of salvage endoscopic submucosal dissection for Barrett's neoplasia recurrence after radiofrequency ablation. Endoscopy 2024; 56: 653-662. doi:10.1055/a-2307-6949
60. Meulen LWT, Bogie RMM, Siersema PD et al. Standardised training for endoscopic mucosal resection of large non-pedunculated colorectal polyps to reduce recurrence (*STAR-LNPCP study): a multicentre cluster randomised trial. Gut 2024; 73: 741-750. doi:10.1136/gutjnl-2023-330020
61. Mohammed A, Gonzaga ER, Hasan MK et al. Low delayed bleeding and high complete closure rate of mucosal defects with the novel through-the-scope dual-action tissue clip after endoscopic resection of large nonpedunculated colorectal lesions (with video). Gastrointest Endosc 2024; 99: 83-90.e81. doi:10.1016/j.gie.2023.07.025
62. Monino L, Marique L, Deswysen Y et al. Endoscopic Management of Biliary and Pancreatic Pathologies in Roux-en-Y Gastric Bypass Patients: Development of a Treatment Algorithm Based on 9-Year Experience. Obes Surg 2024; 34: 3717-3725. doi:10.1007/s11695-024-07471-3
63. Moon JH, Park SW, Lee YN et al. A comparison of novel electrocautery-enhanced lumen-apposing metal stents and plastic stents in EUS-guided drainage of infected walled-off necrosis: A multicenter randomized study. Endoscopy 2024; 56: 926-937. doi:10.1055/a-2342-1140
64. de Moura DTH, Baroni LM, Bestetti AM et al. Evaluation of quality indicators of screening colonoscopy performed in a private quarternary hospital in Brazil. ABCD Arquivos Brasileiros de Cirurgia Digestiva (São Paulo) 2024; 37: e1815. doi:10.1590/0102-6720202400022e1815

Endosc Int Open | DOI 10.1055/a-2965-6729 | © The Author(s).

65. Nimri F, Ichkhanian Y, Shinn B et al. Comprehensive analysis of adverse events associated with transmural use of LAMS in patients with liver cirrhosis: International multicenter study. Endosc Int Open 2024; 12: E740-E749. doi:10.1055/a-2312-1528
66. Pérez-Cuadrado-Robles E, Phillpotts S, Bronswijk M et al. PERcutaneous transhepatic CHOLangioscopy using a new single-operator short cholangioscope (PERCHOL): European feasibility study. Dig Endosc 2024; 36: 719-725. doi:10.1111/den.14697
67. Rancatore G, Ligresti D, Rizzo GEM et al. Endoscopic Ultrasound-Guided Fine Needle Biopsy of Focal Liver Lesions: An Effective Mini-Invasive Alternative to the Percutaneous Approach. Diagnostics (Basel) 2024; 14: 1336. doi:10.3390/diagnostics14131336
68. Rodriguez de Santiago E, van Tilburg L, Deprez PH et al. Western outcomes of circumferential endoscopic submucosal dissection for early esophageal squamous cell carcinoma. Gastrointest Endosc 2024; 99: 511-524. e516. doi:10.1016/j.gie.2023.10.042
69. Roskovicova V, Katuchova J, Madarova N et al. Risk factors for post-ERCP complications. Bratisl Lek Listy 2024; 125: 554-550. doi:10.4149/BLL_2024_85
70. Ruiz-Manriquez J, Olivas-Martinez A, Terán-Ellis SMy et al. Complications in Patients with Surgically Altered Gastrointestinal Anatomy Undergoing Endoscopic Retrograde Cholangiopancreatography: 15-Year Experience at a Tertiary Care Center in Latin America. Dig Dis Sci 2024; 69: 3021-3028. doi:10.1007/s10620-024-08516-4
71. Sadeghi A, Arabpour E, Movassagh-Koolankuh S et al. Primary Needle-Knife Fistulotomy Versus Standard Transpapillary Technique for Cannulation of Long-Size Papilla: A Randomized Clinical Trial. Clin Transl Gastroenterol 2024; 15: e00788. doi:10.14309/ctg.0000000000000788
72. Sadeghi A, Arabpour E, Rastegar R et al. Impact of major duodenal papilla morphology on the outcomes of primary needle-knife fistulotomy for deep biliary cannulation. Sci Rep 2024; 14: 31949. doi:10.1038/s41598-024-83446-9
73. Sanavio M, Vauquelin B, Picot M-C et al. Selective inner muscle layer myotomy is associated with lower pain and same clinical efficacy that full-thickness myotomy in patients treated by POEM for achalasia: a multicenter retrospective comparative analysis of 158 patients. Clin Res Hepatol Gastroenterol 2024; 48: 102401. doi:10.1016/j.clinre.2024.102401
74. Steinbrück I, Ebigbo A, Kuellmer A et al. Cold Versus Hot Snare Endoscopic Resection of Large Nonpedunculated Colorectal Polyps: Randomized Controlled German CHRONICLE Trial. Gastroenterology 2024; 167: 764-777. doi:10.1053/j.gastro.2024.05.013
75. Steinbrück I, Rempel V, Kuellmer A et al. Flexible endoscopic treatment of Zenker's diverticulum—a retrospective, observational multicenter study. Surg Endosc 2024; 38: 6493-6505. doi:10.1007/s00464-024-11234-7
76. Stigliano S, Marocchi G, Baldaro F et al. Timing of lumen-apposing metal stents removal in pancreatic fluid collections: Could we go beyond? Pancreatology 2024; 24: 1252-1256. doi:10.1016/j.pan.2024.10.011
77. Tejedor-Tejada J, Alvarez-Alvarez A, Olmos JM et al. Secondary uncovered versus fully-covered metal stents for the management of occluded stent in unresectable distal malignant biliary obstruction. Gastroenterol Hepatol 2024; 47: 502218. doi:10.1016/j.gastrohep.2024.502218
78. Van Severen C, Koch S, Faure J et al. Peroral endoscopic myotomy: a two-center retrospective study of practice and adverse events. Acta Gastroenterol Belg 2024; 87: 7-13. doi:10.51821/87.1.12358
79. Vargas-Madrigal J, Chan SM, Dhar J et al. Dedicated cautery-enhanced tubular self-expandable metal stent for endoscopic ultrasound-guided hepaticogastrostomy: feasibility study. Endoscopy 2024; 56: 864-869. doi:10.1055/a-2340-7432
80. Vauquelin B, Quénéhervé L, Pioche M et al. Factors associated with early failure of peroral endoscopic myotomy in achalasia. Gastrointest Endosc 2024; 99: 349-357.e343. doi:10.1016/j.gie.2023.10.025
81. Velasquez-Rodriguez JG, Loras C, Maisterra S et al. Effectiveness of endoscopic ultrasound-guided simple puncture-aspiration (non-stenting) in the management of abdominal

collections. Ther Adv Gastrointest Endosc 2024; 17: 26317745241287319. doi:10.1177/26317745241287319
82. Verhoeve S, Verloop C, Bruno M et al. Efficacy of EUS-guided keyhole biopsies in diagnosing subepithelial lesions of the upper gastrointestinal tract. Endosc Int Open 2024; 12: E1183-e1189. doi:10.1055/a-2417-0580
83. Wang Y, Ren Z, Shen Y-H et al. Long-term outcomes of endoscopic resection for well-differentiated nonampullary duodenal neuroendocrine tumors. Gastrointest Endosc 2024; 100: 481-491.e486. doi:10.1016/j.gie.2024.02.017
84. Wessels EM, Nullens S, Bastiaansen BA et al. Routine esophagram to detect early esophageal leakage after peroral endoscopic myotomy. Endosc Int Open 2024; 12: E604-E612. doi:10.1055/a-2294-8607
85. Wilson N, Abdallah M, Schulman AR et al. Outcomes of cold snare EMR of nonampullary duodenal adenomas ≥ 1 cm: a multicenter study. Gastrointest Endosc 2024; 99: 971-980.e971. doi:10.1016/j.gie.2023.12.007
86. Yang D, Bhatt A, Hayat M et al. Novel hemostatic adhesive powder to prevent delayed bleeding after endoscopic submucosal dissection in the GI tract: first U.S. multicenter experience. iGIE 2024; 3: 501-506. doi:10.1016/j.igie.2024.10.002
87. Yang D, Hasan MK, Fawwaz BB et al. Quantification of interstitial cells of Cajal and fibrosis during gastric per-oral endoscopic myotomy and its association with clinical outcomes. Endosc Int Open 2024; 12: E585-E592. doi:10.1055/a-2290-0502
88. Yang D, Hasan MK, Xiao Y et al. The use of a self-assembling peptide gel for stricture prevention in the esophagus after endoscopic submucosal dissection: a US multicenter prospective study (with video). Gastrointest Endosc 2024; 100: 213-220. doi:10.1016/j.gie.2024.03.012
89. Balducci D, Ratone JP, Schaefer M et al. EUS-guided hepaticojejunostomy in patients with history of total gastrectomy: a multicenter retrospective feasibility study (with video). Gastrointest Endosc 2025; 101: 117-122. doi:10.1016/j.gie.2024.07.011
90. Beunon C, Debourdeau A, Schaefer M et al. Technical failure of endoscopic ultrasound choledocoduodenostomy: multicenter case-control study on rescue techniques, consequences and risk factors. Endoscopy 2025; Online ahead of print. doi:10.1055/a-2541-2973
91. Bronswijk M, Gökce E, Hindryckx P et al. Single-session endoscopic ultrasound-directed transgastric endoscopic retrograde cholangiopancreatography with a dedicated over-the-scope fixation device: Feasibility study (with video). Dig Endosc 2025; 37: 176-182. doi:10.1111/den.14879
92. Dahel Y, Caillol F, Ratone J-P et al. Safety of intrabiliary radiofrequency ablation in cases of residual and recurrent neoplasia after endoscopic papillectomy. Endosc Int Open 2025; 13: a24872598. doi:10.1055/a-2487-2598
93. Debourdeau A, Daniel J, Caillo L et al. Effectiveness of endoscopic ultrasound (EUS)-guided choledochoduodenostomy vs. EUS-guided gallbladder drainage for jaundice in patients with malignant distal biliary obstruction after failed endoscopic retrograde cholangiopancreatography: Retrospective, multicenter study (GALLBLADEUS Study). Dig Endosc 2025; 37: 103-114. doi:10.1111/den.14750
94. Fortunati F, Monino L, Deprez P et al. Endoscopic retrograde cholangiopancreatography in patients with different types of total and partial gastrectomy. Acta Gastroenterol Belg 2025; 88: 23-30. doi:10.51821/88.1.13779
95. Garg R, Baron T, Trieu JA et al. Safety and Effectiveness of EUS-Guided Gallbladder Drainage in Patients with Cirrhosis: An International Multicenter Experience. Endoscopy 2025; 57: 593-601. doi:10.1055/a-2517-0927
96. Gökce E, Devisscher L, Rashidian N et al. Lumen-apposing metal stents for anastomosis creation throughout the gastrointestinal tract: A large single-center experience. DEN open 2025; 5: e419. doi:10.1002/deo2.419

97. Gonzalez J-M, Phelip J, Gasmi M et al. Endoscopic Ultrasound Guided Gastroenterostomy for managing gastroparesis refractory to gastric peroral endoscopic pylorotomy: a new promising therapeutic option. iGIE 2025; 4: 16-20. doi:10.1016/j.igie.2025.01.011
98. Ichkhanian Y, Chaudhary AJ, Veracruz N et al. Endoscopic Ultrasound-Guided Drainage of Intra-Abdominal Abscess Using 15-mm vs. 10-mm Lumen-Apposing Metal Stents: An International Case-Matched Study. Gastrointest Endosc 2025; Online ahead of print. doi:10.1016/j.gie.2025.01.002
99. Kadkhodayan K, Pathak S, Chandan S et al. EUS-guided rendezvous is a viable salvage technique for failed billiary cannulation in patients with Roux-en-Y gastric bypass undergoing BAE-ERCP. Endosc Int Open 2025; 13: a25097500. doi:10.1055/a-2509-7500
100. Lee H, Park KH, Rosas U et al. Predictive factors for early mortality after EUS-guided gastroenterostomy in malignant gastric outlet obstruction. Endosc Int Open 2025; 13: a24749802. doi:10.1055/a-2474-9802
101. Lisotti A, D'Errico F, Fusaroli P et al. Safety and efficacy of endoscopic ultrasound-guided pelvic abscess drainage with lumen-apposing metal stents for complicated acute diverticulitis. Gastrointest Endosc 2025; Online ahead of print. doi:10.1016/j.gie.2025.02.003
102. Martinez-Moreno B, Martínez JM, Herrera I et al. Correlation of endoscopic ultrasound-guided portal pressure gradient measurements with hepatic venous pressure gradient: a prospective study. Endoscopy 2025; 57: 62-67. doi:10.1055/a-2369-0759
103. Mega PF, de Moura EGH, Bestetti AM et al. Endoscopic Vacuum Therapy for the Management of Non-Variceal Upper Gastrointestinal Bleeding: A Valuable Resource for the Endoscopist’s Toolbox. Endoscopy 2025; Online ahead of print. doi:10.1055/a-2544-6448
104. Neri B, Stigliano S, Biasutto D et al. Endoscopic ultrasound-guided entero-colostomy with lumen-apposing metal stent as a rescue treatment for malignant intestinal occlusion: a multicenter study. Endoscopy 2025; 57: 77-82. doi:10.1055/a-2354-3352
105. Ogura T, Ueno S, Okuda A et al. Technical outcomes between a drill dilator and ultra-tapered mechanical dilator during EUS-guided pancreaticogastrostomy: Comparative study. Endoscopy International Open 2025; 13: a24873545. doi:10.1055/a-2487-3545
106. Pollack E, Norwood D, Caceres H et al. Lumen-apposing metal stents provide early and late clinical benefits for the management of benign gastrointestinal strictures: Is there a role for definitive therapy? DEN open 2025; 5: e70005. doi:10.1002/deo2.70005
107. Romanczyk M, Klimkowski R, Kukla M et al. Safety, efficacy and changing trends in endoscopic ablation for dysplastic Barrett’s esophagus in Poland: a long-term multicenter retrospective analysis. Pol Arch Intern Med 2025; 135: 16912. doi:10.20452/pamw.16912
108. Salame M, Ichkhanian Y, Hadaki N et al. Safety of Same-Day Discharge After Gastric Peroral Endoscopic Myotomy in Patients with Refractory Gastroparesis: An International Multi-Center Study. Gastrointest Endosc 2025; Online ahead of print. doi:10.1016/j.gie.2025.02.020
109. Sarkis Y, Stainko S, Perkins A et al. Comparison of flexible endoscopic needle-knife septotomy and peroral endoscopic myotomy for treatment of Zenker's diverticulum. Gastrointest Endosc 2025; 101 82-89. doi:10.1016/j.gie.2024.08.035
110. Shiomi H, Saito T, Hamada T et al. Quadrant, necrosis, and infection criteria for the risk stratification of walled-off necrosis: external validation using multi-institutional data. Gastrointest Endosc 2025; 102: 67-78.e63. doi:10.1016/j.gie.2024.12.037
111. Tachi K, Hara K, Okuno N et al. Efficacy and safety of endoscopic ultrasound-guided hepaticogastrostomy for biliary drainage in hypervascular hepatocellular carcinoma. Clin Endosc 2025; 58: 448-456. doi:10.5946/ce.2024.079
112. Terrin M, D’Errico F, Rotkopf H et al. First-intention EUS-guided transluminal drainage with LAMS: an effective and safe method for management of fluid collections after any kind of surgery. Surg Endosc 2025; 39: 2415-2424. doi:10.1007/s00464-025-11615-6
113. Wannhoff A, Canakis A, Sharaiha RZ et al. Endoscopic ultrasound-guided gastroenterostomy for the treatment of gastric outlet obstruction secondary to acute pancreatitis. Endoscopy 2025; 57: 249-254

Endosc Int Open | DOI 10.1055/a-2965-6729 | © The Author(s).

114. Nass KJ, Zwager LW, van der Vlugt M et al. Novel classification for adverse events in GI endoscopy: the AGREE classification. Gastrointest Endosc 2022; 95: 1078-1085 e1078. doi:10.1016/j.gie.2021.11.038
115. Cotton PB, Eisen GM, Aabakken L et al. A lexicon for endoscopic adverse events: report of an ASGE workshop. Gastrointest Endosc 2010; 71: 446-454. doi:10.1016/j.gie.2009.10.027
116. Dindo D, Demartines N, Clavien PA. Classification of surgical complications: a new proposal with evaluation in a cohort of 6336 patients and results of a survey. Ann Surg 2004; 240: 205-213. doi:10.1097/01.sla.0000133083.54934.ae
117. Clavien PA, Barkun J, de Oliveira ML et al. The Clavien-Dindo classification of surgical complications: five-year experience. Ann Surg 2009; 250: 187-196. doi:10.1097/SLA.0b013e3181b13ca2

Endosc Int Open | DOI 10.1055/a-2965-6729 | © The Author(s).
